# Quantitative interpretation models for targeted next-generation sequencing in lower respiratory tract infections: a multicenter prospective study

**DOI:** 10.1186/s12931-026-03690-7

**Published:** 2026-05-09

**Authors:** Chuwei Jing, Yuchen Ding, Ji Zhou, Jiachen Wei, Mingyue Wang, Dongmei Yuan, Liangfei Peng, Youming Huang, Xuefei Shi, Xiaodong Wu, Lili Tao, Qian Qian, Wenkui Sun

**Affiliations:** 1 Department of Respiratory and Critical Care Medicine, Jiangsu Province Hospital/Nanjing Medical University First Affiliated Hospital, Nanjing, Jiangsu 210029 China; 2 Jiangsu Health Vocational College, Nanjing, Jiangsu China; 3 Department of Respiratory Medicine, Jinling Hospital, Nanjing University School of Medicine, Nanjing, China; 4 Department of Respiratory and Critical Care Medicine, The Second Affiliated Hospital of Wannan Medical College, Wuhu, Anhui Province China; 5 Department of Respiratory Medicine, Huzhou Central Hospital, Fifth School of Clinical Medicine of Zhejiang Chinese Medical University, Huzhou, China; 6 Department of Respiratory and Critical Care Medicine, Shanghai East Hospital, Tongji University, Shanghai, China; 7 Vanderbilt University Medical Center, Nashville, TN USA

**Keywords:** Lower respiratory tract infection, Targeted next-generation sequencing, RPKM, Copy number, Nomogram, Pathogen detection, Antimicrobial resistance genes, Diagnostic model

## Abstract

**Background:**

Lower respiratory tract infections (LRTIs) represent a significant global health burden. While targeted next-generation sequencing (tNGS) offers potential advantages for pathogen detection, its clinical implementation is hindered by the absence of validated quantitative interpretation criteria for pathogen discrimination.

**Methods:**

We conducted a multicenter prospective study of 631 patients with suspected LRTIs across five intensive care units in eastern China from January 2022 to March 2025. Bronchoalveolar lavage fluid specimens underwent concurrent tNGS and conventional microbiological testing (CMT). Expert group A established the reference standard by classifying patients into LRTI/non-LRTI categories and identifying clinically significant pathogens based on comprehensive clinical criteria. Expert group B, blinded to tNGS quantitative data, provided qualitative interpretation based solely on detected microorganisms to eliminate any influence from quantitative parameters. Expert group C, blinded to all tNGS data, provided interpretation based on conventional microbiological testing combined with clinical manifestations. Quantitative diagnostic models incorporating reads per kilobase per million mapped reads (RPKM) and pathogen copy numbers were developed using a training cohort (*n* = 420) and validated in an independent cohort (*n* = 211).

**Results:**

Of 631 patients, 358 (56.7%) met the diagnostic criteria for LRTI. Polymicrobial infections were identified in 77 patients, with the majority co-infected with *Acinetobacter baumannii* and *Pseudomonas aeruginosa*. tNGS demonstrated enhanced detection of Gram-negative bacteria, *Candida* species and *Pneumocystis jirovecii*, while CMT showed better detection for *Aspergillus* species. The quantitative models demonstrated excellent discriminatory performance for bacterial pathogens. The sensitivity and specificity for conventional microbiological testing alone were 58.7% and 74.7%. Adding clinical manifestations to CMT resulted in a sensitivity of 68.8% and specificity of 72.0%. In comparison, qualitative tNGS achieved a sensitivity of 78.5% and a specificity of 76.6%, while the model-based algorithm demonstrated the highest diagnostic accuracy with a sensitivity of 82.4% and a specificity of 85.0%. For antimicrobial resistance prediction, tNGS achieved moderate accuracy (AUC 0.715) with high concordance for key antimicrobial resistance markers including KPC, NDM, OXA-48 and mecA.

**Conclusion:**

We developed and validated quantitative models for tNGS-based pathogen detection in LRTIs, enabling precise discrimination between pathogenic and background organisms. These models represent a significant step forward in the clinical application of tNGS for LRTI diagnosis and antimicrobial resistance detection.

**Supplementary Information:**

The online version contains supplementary material available at 10.1186/s12931-026-03690-7.

## Introduction

Lower respiratory tract infections (LRTIs) are caused by a diverse array of pathogens and constitute the fourth leading cause of global mortality [1, 2]. Effective treatment of LRTIs relies on the precise identification of causative pathogens [3]. However, conventional microbiological tests (CMTs) have several limitations, including prolonged turnaround times of 48–72 h, difficulty in distinguishing infection from background microbiota, low sensitivity for detecting fastidious pathogens and reduced specificity following prior antibiotic exposure [4].

Metagenomic next-generation sequencing (mNGS) has revolutionized pathogen detection by enabling the unbiased sequencing of all nucleic acids present in clinical samples. Despite its potential, clinical implementation of mNGS is limited by high costs [5], complex bioinformatic requirements [6], and vulnerability to contamination [7]. Targeted next-generation sequencing (tNGS) represents an alternative approach that utilizes ultra-multiplex PCR amplification to detect preselected genomic regions from 153 respiratory pathogens (Table S2) responsible for over 95% of respiratory infections [8] and 15 antimicrobial resistance genes (Table S3). This targeted strategy minimizes both cost and the complexity of bioinformatic analysis [9]. Beyond pathogen detection, tNGS provides quantitative insights through two complementary metrics: RPKM and pathogen DNA/RNA copy number. RPKM stands for reads per kilobase of genome/transcript per million reads mapped. RPKM is a normalized unit of transcript abundance commonly used in RNA-seq and targeted sequencing. This normalization accounts for both sequencing depth and gene length, allowing direct comparison of abundance across different features within a sample or between samples. The copy number of a pathogen refers to the absolute or relative quantity of its nucleic acid detected in a clinical sample [10]. Based on our previous research [11], we developed a semiquantitative grading system adapted from traditional microbiological culture methods to tackle the challenges in quantifying high-magnitude pathogen copy numbers. Pathogen copy numbers were categorized into four levels, namely, < 1 × 10^4^ copies (level 1), 1 × 10⁴ to < 1 × 10⁵ copies (level 2), 1 × 10⁵ to < 1 × 10⁶ copies (level 3), and ≥ 1 × 10⁶ copies (level 4). Despite its quantitative capabilities, the interpretation of tNGS results remains challenging, often relying on subjective assessments by multidisciplinary teams consisting of microbiologists, clinicians, and bioinformaticians. This introduces considerable inter-observer variability in sensitivity and specificity [12, 13], thereby hindering broader clinical implementation. While RPKM and copy number metrics provide objective analytical frameworks for standardizing interpretation protocols, there is currently no comprehensive large-scale study that has defined evidence-based threshold criteria using data from clinical cohorts.

This multicenter blinded prospective study aimed to develop and validate quantitative interpretation models for pathogen detection in LRTIs based on tNGS-derived RPKM and copy numbers. Our primary objectives included developing pathogen-specific nomogram models using a training cohort, followed by validation in an independent cohort. We subsequently compared the diagnostic performance of model-guided tNGS interpretation to CMTs, using expert consensus as the reference. Additionally, we evaluated the capability of tNGS to predict antimicrobial resistance. This represents the first systematic effort to transform tNGS from a qualitative detection tool to a quantitative diagnostic platform with standardized clinical interpretation criteria.

## Methods

### Study design and patient population

**Fig. 1:**
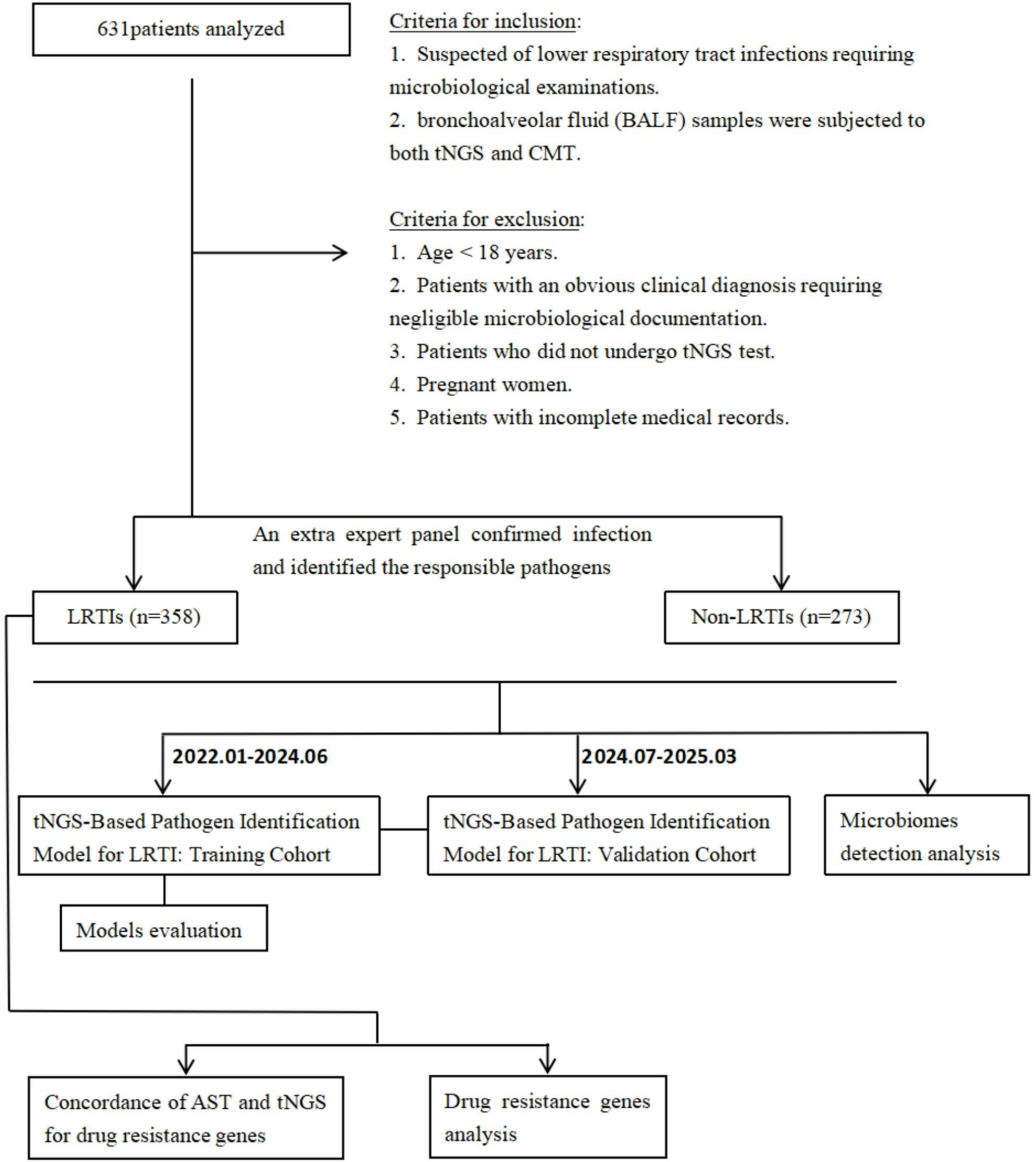
Flowchart of patient enrollment, classification, and comparison. BALF was collected and simultaneously submitted for CMT and tNGS. Diagnostic value of these tests was assessed

This prospective cohort study enrolled 631 patients with suspected lower respiratory tract infections (LRTIs) from five Intensive Care Units (ICU) in eastern China between January 2022 and March 2025 (Fig. 1, Table S1). Five ICUs were from five independent hospitals. CMT was performed independently at each participating hospital’s clinical microbiology laboratory. The core components of CMT, including Gram staining, bacterial/fungal culture, Aspergillus galactomannan testing, and M. tuberculosis PCR, were harmonized based on the National Clinical Laboratory Procedures (4th edition) issued by the Chinese Ministry of Health [14]. Each center’s clinical microbiology laboratory was certified and underwent regular external quality assessment (EQA) by the National Center for Clinical Laboratories.

Patients were enrolled in the study if they met the following criteria: (1) suspected of LRTI requiring microbiological testing; (2) bronchoalveolar lavage fluid (BALF) samples were collected and submitted for both tNGS and CMT. Patients were suspected of having a LRTI when they fulfilled all the following criteria [15]: (1) at least one compatible symptom, such as new-onset fever, cough, or dyspnea; (2) new-onset radiological findings on chest imaging; or (3) treatment with empirical antibiotics prior to ICU admission or within 48 h of being transferred to the ICU. Exclusion criteria were as follows: (1) Age < 18 years; (2) Patients with an obvious clinical diagnosis requiring negligible microbiological documentation; (3) Patients who did not undergo tNGS testing; (4) Pregnant women; (5) Patients with incomplete medical records. This study was approved by the Institutional Review Board and Ethics Committee of Nanjing Medical University (approval no. 2023-SR-161) and conducted in accordance with the Declaration of Helsinki.

The study population was divided into two cohorts: a training cohort, consisting of 420 patients (January 2022 to June 2024) for model development and a validation cohort, consisting of 211 patients (July 2024 to March 2025) for model validation. These cohorts were analyzed separately to ensure robust model performance evaluation.

### Data collection

Comprehensive patient information, including demographic characteristics, clinical parameters, laboratory findings, radiological results, and microbiological data were collected by a dedicated data collection team throughout a 30-day follow-up period.

### Conventional microbiological tests

All BALF specimens from enrolled patients were submitted for CMT, which included Gram staining, bacterial smear and culture, fungal smear and culture, *Aspergillus* galactomannan testing, and *Mycobacterium tuberculosis* DNA detection. The CMT results were retrieved from the hospital’s electronic medical record system.

### tNGS of BALF: laboratory processing and bioinformatics

#### Sample preparation

BALF samples were independently collected at each participating hospital. All five ICUs followed the same BALF collection procedures described in our prior study [16]. Samples were stored on dry ice before being dispatched to the NanJing Kingmed Diagnostics Laboratory Co., Ltd. for further tNGS analysis within 8 h after collection [17]. Positive and negative controls from a respiratory pathogen detection kit (KS608-100HXD96, KingCreate, Guangzhou, China) were included to monitor the entire tNGS process. Each 650-µL BALF sample was liquefied with an equal volume of 80 mmol/L dithiothreitol and homogenized for 15 s using a vortex mixer. Total nucleic acid extraction and purification were performed on 500 µL of homogenate using a MagPure pathogen DNA/RNA kit (R6672-01B, Magen, Guangzhou, China) following the manufacturer’s protocol.

#### Library construction and sequencing

Libraries were prepared using the respiratory pathogen detection kit with non-template controls included. The process involved two rounds of PCR amplification using extracted nucleic acid and cDNA as templates. Ultra-multiplex PCR amplification was performed with 153 microorganism-specific primers (Supplementary Table S2) targeting bacteria, viruses, fungi, mycoplasma, and chlamydia.

Following amplification, PCR products were purified using magnetic beads and re-amplified with primers containing sequencing adapters and unique barcodes. Library quality and quantity were assessed using a Qsep100 Bio-Fragment analyzer (Bioptic, Taiwan, China) and Qubit 4.0 fluorometer (Thermo Scientific, Massachusetts, USA), respectively. Library fragments typically ranged from 250 to 350 bp with concentrations at ≥ 0.5 ng/µL.

The pooled library was diluted to a concentration of 1 nmol/L, and 5 µL was mixed with 5 µL of freshly prepared 0.1 mol/L NaOH. After brief vortexing and centrifugation, the mixture was incubated at room temperature for 5 min. Sequencing was then performed on an Illumina MiniSeq platform using a universal sequencing reagent kit (KS107-CXR, KingCreate), generating approximately 0.1 million single-end 100 bp reads per library.

### Bioinformatics analysis

Sequencing data were analyzed using a data management and analysis system 3.7.2 (KingCreate). Adapters were identified and trimmed from the raw sequencing reads. Reads with single-end lengths exceeding 50 bp were retained, and were sent to low-quality filtering to retain reads with Q30 > 75%, ensuring high-quality data. The single-ended aligned reads were then compared using the Self-Building clinical pathogen database to determine the read count of specific amplification targets in each sample. The reference sequences used for read mapping were cited from a database curated from different sources, including Genbank database, Refseq database, and Nucleotide database from National Center for Biotechnology Information (NCBI).

### Bioinformatic thresholds for tNGS microbial identification

The quantitative performance of tNGS has been validated in our previous research [11]. In line with the experimental principle of targeted amplification of microbial sequences using specific primers, the amplicon coverage and normalized read count of the detected microorganisms within the sample constituted the primary interpretation indicators. To categorize a microorganism as a potential pathogen, three criteria were established: (i) Amplicon coverage was ≥ 50% and normalized read count was ≥ 10 for bacteria (excluding Mycobacterium tuberculosis complex), fungi and atypical pathogens. (ii) Amplicon coverage was ≥ 50%, and normalized read count was ≥ 3, or normalized read count was ≥ 10 for viruses. (iii) Normalized read count was ≥ 1 for Mycobacterium tuberculosis complex.

### Identification of clinically significant pathogens for LRTI

Expert group A, consisting of two intensivists with expertise in managing LRTI (WKS and JZ), independently reviewed the medical records of all patients, including results of CMTs and tNGS. They first determined whether the patients had infectious or noninfectious aetiology. They then identified ‘clinically significant pathogens’ based on clinical manifestation, laboratory tests, chest radiology, microbiological tests (including CMTs and tNGS), and treatment response. Any disagreement between the two intensivists was resolved by in-depth discussion, and another senior intensivist (Dongmei Yuan) was consulted if consensus could not be reached.

### Development and validation of clinically significant pathogen diagnostic model using tNGS-derived RPKM and pathogen copy number

Using reference pathogens identified above, we developed diagnostic models incorporating tNGS-derived RPKM values and pathogen copy numbers. To tackle the challenges in quantifying high-magnitude pathogen copy numbers, we developed a semiquantitative grading system adapted from traditional microbiological culture methods. Pathogen copy numbers were categorized into four levels: <1 × 10⁴ copies (level 1), 1 × 10⁴ to < 1 × 10⁵ copies (level 2), 1 × 10⁵ to < 1 × 10⁶ copies (level 3), and ≥ 1 × 10⁶ copies (level 4).

For pathogens meeting the predefined tNGS detection thresholds (refer to ‘Bioinformatic Thresholds for tNGS Microbial Identification’ section above), diagnostic models were developed to predict their clinical relevance. Binary logistic regression analysis was performed to develop a diagnostic model for clinically significant pathogen detection based on tNGS results using the training cohort of 420 patients enrolled from January 2022 to June 2024. The model incorporated both RPKM and pathogen copy number as primary predictors. The optimal probability cut-off for clinically significant pathogen diagnosis was determined by maximizing the Youden index (sensitivity + specificity − 1) in the training cohort.

Model performance was evaluated using multiple metrics in the training cohort. Receiver operating characteristic (ROC) curves were generated to assess discriminative ability, with area under the curve (AUC) values interpreted as follows: 0.5–0.7 (low predictive value), 0.7–0.9 (moderate predictive value), and > 0.9 (high predictive value). Model calibration was assessed using calibration plots and the Hosmer-Lemeshow goodness-of-fit test. Decision curve analysis (DCA) was performed to evaluate the clinical utility of the model by quantifying standardized benefit across different probability thresholds.

The final logistic regression model generated a probability equation for determining whether a specific pathogen is clinically significant, incorporating both RPKM values and pathogen copy number levels. The equation allows calculation of the probability of clinical relevance for individual pathogens in each patient using their specific tNGS-derived parameters.

The diagnostic model was validated using the validation cohort (*n* = 211, July 2024 to March 2025). Model discrimination was assessed using C-index values and ROC curves, with C-index interpretations as follows: 0.7–0.8 (acceptable discrimination), 0.8–0.9 (very good discrimination), and > 0.9 (outstanding discrimination). The established probability equation was applied to calculate the predicted probabilities of clinical relevance for specific pathogens in all patients in the validation cohort using their RPKM values and pathogen copy number levels. Predicted outputs were compared with clinically significant pathogens diagnoses to calculate sensitivity, specificity, positive predictive value, negative predictive value, and overall accuracy. The Hosmer-Lemeshow goodness-of-fit test was performed with 5 probability groups based on sample size considerations. This grouping strategy ensured ≥ 40 observations per group as recommended for reliable χ² approximation.

### Comparative analysis of tNGS and CMT

To specifically evaluate the influence of tNGS quantitative data (RPKM, copy number levels) on the adjudication of clinical relevance by experts, two additional experienced intensivists (Expert Group B, consisting of LFP and YMH), blinded to the tNGS quantitative data and the model predictions, independently reviewed the same patient data as Expert Group A. They were provided only with the ‘qualitative’ list of pathogens detected by tNGS and all CMT results. Their adjudication of clinical relevance for each pathogen followed the same principles as Expert Group (A) Disagreements were resolved similarly. The assessments by Expert Group B served to establish the reference standard adjudication without the potential bias introduced by quantitative tNGS data, allowing comparison with the primary reference standard (Expert Group A) which had access to quantitative tNGS data. Expert group C, consisting of two intensivists with expertise in managing LRTI (XDW and QQ), blinded to all tNGS data, independently reviewed the same patient data as Expert Group A and (B) The assessments by Expert Group C completely eliminated the incorporation bias of tNGS. We evaluated and compared four diagnostic categories: conventional microbiological testing alone, conventional microbiological testing combined with clinical manifestations, qualitative targeted next-generation sequencing, and model-based targeted next-generation sequencing. The definition of clinical manifestations in our study is the integrated assessment performed by senior clinicians based on a comprehensive review of clinical symptoms, imaging features, and the patient’s response to treatment.

Pathogen-Level Comparison: optimal probability cut-offs, derived from the diagnostic models incorporating RPKM values and copy number levels, were used to define the criteria for classifying a tNGS-detected pathogen as ‘clinically significant’ in subsequent analyses. For each reference pathogen identified by Expert Group A, the detection abilities of tNGS (model-guided), CMT alone, CMT combined with clinical manifestations, and qualitative tNGS were compared. Viral pathogens were excluded from the analysis due to their preferential tropism for the upper respiratory tract and limited detection cases in bronchoalveolar lavage fluid (BALF) in our cohort.

Patient-Level Comparison: particularly in polymicrobial infections, a patient was considered positive by a method (tNGS or CMT) if all clinically significant pathogens identified by the expert group A were detected. Failure to detect at least one such pathogen rendered the patient-level result negative. Diagnostic performance metrics (sensitivity, specificity, PPV, NPV, accuracy) at the patient level were calculated and compared between tNGS and CMT.

### Antimicrobial resistance gene analysis

The concordance between the presence of antimicrobial resistance (AMR) genes detected by tNGS and the phenotypic antimicrobial susceptibility testing (AST) results obtained from CMT (for corresponding bacterial isolates) was assessed. Genotype-phenotype concordance was defined as the detection of an AMR gene by tNGS corresponding to the phenotypic resistance profile observed in AST for the significant antimicrobial class(es). In the concordance analysis, we applied a strict data-driven approach to attribute detected ARGs to specific bacterial hosts. First, for samples containing multiple potential host pathogens, we used phenotypic AST results as the reference. If tNGS detected an ARG (e.g. NDM) together with multiple species, the ARG was attributed only to the pathogen that demonstrated phenotypic resistance to the corresponding drug class in the AST report. Second, when multiple co-detected pathogens exhibited the same resistance phenotype (e.g., both K. pneumoniae and Acinetobacter were carbapenem-resistant), making definitive attribution impossible using short-read sequencing, such cases were excluded from the species-level analysis. Third, for highly host-specific genes, such as mecA in S. aureus, the attribution was made directly based on known biological associations.

### Statistical analysis

Statistical analyses were performed using SPSS version 25.0 (IBM Corp., Armonk, NY, USA) and R version 4.3.1 (R Foundation for Statistical Computing, Vienna, Austria). Continuous variables are presented as medians (interquartile ranges) and categorical variables as frequencies (percentages). Proportions were compared using chi-square or Fisher’s exact tests. Pathogen detection rates and agreement between tNGS and CMT were assessed using paired chi-square tests and kappa statistics, respectively. To account for potential clustering of patients within study centers, we performed a sensitivity analysis using a cluster-robust variance model. Using the robcov function in the rms package (R version 4.3.1), we specified ‘Center_ID’ as the clustering variable to obtain robust standard errors and confidence intervals. This approach accounts for intra-center correlation while maintaining a single population-averaged model, which is essential for the construction of a clinically applicable nomogram. The results of this cluster-robust analysis are presented in Supplementary Table S4.

## Results

### Patient characteristics

The study included a total of 631 patients with suspected lower respiratory tract infections (LRTIs), of whom 358 (56.7%) were confirmed as LRTI cases, while 273 (43.3%) served as non-LRTI controls. LRTI patients demonstrated significantly elevated inflammatory markers compared to controls, including leukocytes (9.51 vs. 8.48 × 10⁹/L, *p* = 0.004), neutrophils (8.20 vs. 6.84 × 10⁹/L, *p* < 0.001), C-reactive protein (89.39 vs. 54.05 mg/L, *p* < 0.001), procalcitonin (0.49 vs. 0.29 µg/L, *p* < 0.001), and interleukin-6 (43.48 vs. 32.56 pg/mL, *p* = 0.001). Clinical outcomes were significantly worse in LRTI patients, with longer hospitalization duration (12 vs. 10 days, *p* = 0.006), extended ICU stay (10 vs. 9 days, *p* < 0.001), and higher in-hospital mortality (24.86% vs. 16.85%, *p* = 0.015). Additional baseline characteristics are presented in Table S1.

### RPKM and COPY numbers as effective predictors of clinically significant pathogens in LRTI

#### Univariate analysis

Significant differences in RPKM and COPY values of specific pathogens were observed between LRTI and non-LRTI patients (Table 1). The median RPKM and COPY values of major pathogens, such as *Acinetobacter baumannii* and *Klebsiella pneumoniae*, were notably higher in the LRTI group (*p* < 0.001). These findings highlight that elevated RPKM and copy numbers are strongly associated with confirmed lower respiratory tract infections.

**Table 1:** RPKM and COPY of pathogenic bacteria detected by tNGS between LRTI and Non-LRTI patients in the training cohort

Variables	All	LRTI	Non-LRTI	*P* value
RPKM, median (95%CI)	1117(802,1744)	28,310(23046,33247)	297(251,438.07)	< 0.001
COPY, *n*(%[95%CI])				
Level 1	229(42.72% [38.6,46.95])	10(5.75%[3.15,10.25])	219(60.5% [55.38,65.4])	< 0.001
Level 2	94(17.54% [14.55,20.98])	18(10.34%[6.64,15.76])	76(20.99% [17.11,25.48])	
Level 3	41(7.65% [5.69,10.21])	10(5.75%[3.15,10.25])	31(8.56% [6.1,11.9])	
Level 4	172(32.09% [28.28,36.16])	136(78.16%[71.45,83.66])	36(9.94% [7.27,13.46])	
Acinetobacter baumannii				
RPKM, median (95%CI)	4878(2182,23065)	50,699(44706,56888.48)	371(188,802)	< 0.001
COPY, *n*(%[95%CI])				
Level 1	43(32.09% [24.78,40.4])	3(4.62% [1.58,12.71])	40(57.97% [46.21,68.89])	< 0.001
Level 2	24(17.91% [12.34,25.27])	3(4.62% [1.58,12.71])	21(30.43% [20.85,42.08])	
Level 3	8(5.97% [3.06,11.34])	2(3.08% [0.85,10.54])	6(8.7% [4.05,17.7])	
Level 4	59(44.03% [35.91,52.49])	57(87.69% [77.55,93.63])	2(2.9% [0.8,9.97])	
Pseudomonas aeruginosa				
RPKM, median (95%CI)	3430(1015,8226.5)	14320.5(7159.22,19781.3)	411.5(101.5,1277)	< 0.001
COPY, *n*(%[95%CI])				
Level 1	15(24.59% [15.51,36.68])	1(3.23% [0.57,16.19])	14(46.67% [30.23,63.86])	0.001
Level 2	5(8.2% [3.55,17.79])	4(12.9% [5.13,28.85])	1(3.33% [0.59,16.67])	
Level 3	7(11.48% [5.67,21.84])	3(9.68% [3.35,24.9])	4(13.33% [5.31,29.68)	
Level 4	34(55.74% [43.3,67.49])	23(74.19% [56.75,86.3])	11(36.67% [21.87,54.49])	
Klebsiella pneumoniae				
RPKM, median (95%CI)	781.5(285.85,1882)	32,471(24273,46378)	276(115,520)	< 0.001
COPY, *n*(%[95%CI])				
Level 1	51(3.12% [43.22,62.79])	2(7.41% [2.06,23.37])	49(71.01% [59.43,80.38])	< 0.001
Level 2	13(13.54% [8.09,21.8])	2(7.41% [2.06,23.37])	11(15.94% [9.14,26.33])	
Level 3	4(4.17% [1.63,10.23])	-	4(5.8% [2.28,13.98])	
Level 4	28(29.17)	23(85.19% [67.52,94.08])	5(7.25% [3.13,15.87])	

*P* Values were calculated by Mann–Whitney U test

### Collinearity analysis

Variance inflation factor (VIF) analysis demonstrated low collinearity between RPKM and COPY measurements across all pathogens (VIF < 10 for all species), indicating these variables provide independent information (Table 2). This supports their simultaneous inclusion in subsequent multivariable models without significant confounding.

**Table 2:** Collinearity analysis of RPKM and COPY of pathogens detected by tNGS between LRTI and Non-LRTI patients in the training cohort

Pathogen	VIF
*Acinetobacter baumannii*	1.931
*Pseudomonas aeruginosa*	1.076
*Klebsiella pneumoniae*	2.325

*VIF* variance inflation factor

### Development and evaluation of clinically significant pathogens nomogram models for LRTI

**Fig. 2:**
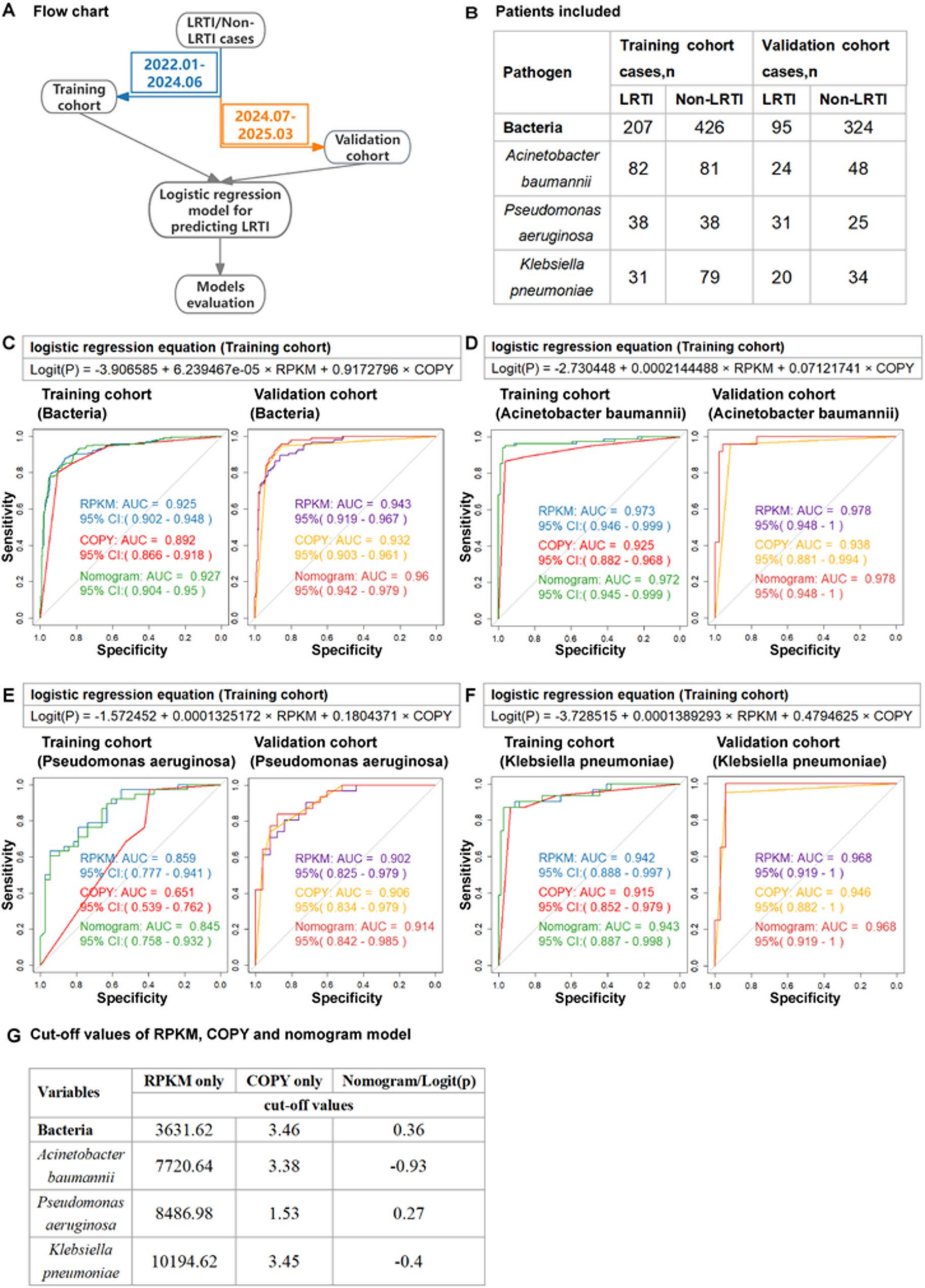
Diagnostic models establishment based on tNGS-derived RPKM and COPY of pathogens. **A** Study design flowchart illustrating the temporal cohorts. **B** Patient stratification by pathogen type (bacteria/fungi) and infection status (LRTI/non-LRTI), demonstrating cohort balance. **C**-**F** Model performance metrics for bacteria overall, Acinetobacter baumannii, Pseudomonas aeruginosa and Klebsiella pneumoniae. **G** Clinically actionable thresholds

Patients enrolled between January 2022 and June 2024 constituted the training cohort, with those enrolled between July 2024 and March 2025 forming the validation cohort (Fig. 2A-B). Multivariable logistic regression incorporating targeted next-generation sequencing (tNGS) derived RPKM and copy number was performed for an overall bacterial model and three specific bacterial models in the training cohort (Fig. 2C-F). The overall bacteria model, utilizing RPKM and copy number values of all bacteria detected by tNGS, was designed to predict whether the detected bacteria were clinically significant pathogens. This model showed an AUC of ROC (AUC-ROC) of 0.927 (95% CI, 0.904–0.950). For individual bacterial species models, the AUC-ROC was 0.972 (95% CI, 0.945–0.999) for *Acinetobacter baumannii*, 0.845 (95% CI, 0.758–0.932) for *Pseudomonas aeruginosa* and 0.943 (95% CI, 0.887–0.998) for *Klebsiella pneumoniae*, respectively. Clinical nomograms were constructed from these models (Figures S2-S5A), with optimal diagnostic thresholds determined by ROC analysis (Fig. 2G).

Model calibration was assessed using the Hosmer-Lemeshow test. The Overall bacterial model demonstrated adequate calibration (χ² = 6.713; *P* = 0 0.082) (Figure S2D). Individual bacterial species also showed good calibration: *Acinetobacter baumannii* (χ² = 5.996; *P* = 0.112) ( Figure S3D), *Pseudomonas aeruginosa* (χ² = 2.134; *P* = 0.545) (Figure S4D), and *Klebsiella pneumoniae* (χ² = 2.601; *P* = 0 0.457) (Figure S5D).

Decision curve analysis demonstrated clinical utility for the overall bacterial model across probability thresholds of 0% to 93% (Figure S2E). Species-specific models showed the clinical utility ranges from 0% to 98% for *Acinetobacter baumannii* (Figure S3E), 0% to 99% for *Pseudomonas aeruginosa* (Figure S4E), and 0% to 99% for *Klebsiella pneumoniae* (Figure S5E). The predictive nomograms have been embedded in an interactive web-based calculator, which can be accessed at https://brownvivian.shinyapps.io/RData/. Detailed instructions for model implementation and interface navigation are provided in Figure S1.

To assess the robustness of our findings to potential clustering effects, we performed a sensitivity analysis using a cluster-robust variance model with study center as the clustering variable. As shown in Supplementary Table S4, both RPKM and copy number remained highly significant predictors (*p* < 0.001) after accounting for intra-center correlation, confirming that our nomogram models are robust to variations between centers.

### Validation of the nomogram models

In the validation cohort, the overall bacterial model maintained excellent discrimination (C-index, 0.926; AUC- ROC, 0.960; 95% CI, 0.942–0.979; Figure S2C). Performance of individual bacterial species remained robust for *Acinetobacter baumannii* (C-index, 0.970; AUC-ROC, 0.978; 95% CI, 0.948-1.000; Figure S3C), *Pseudomonas aeruginosa* (C-index, 0.834; AUC-ROC, 0.914; 95% CI, 0.842–0.985; Figure S4C) as well as for *Klebsiella pneumoniae* (C-index, 0.939; AUC-ROC, 0.968; 95% CI, 0.919-1.000; Figure S5C).

### Comparison of tNGS and CMT for detecting clinically significant pathogens in LRTI

**Fig. 3:**
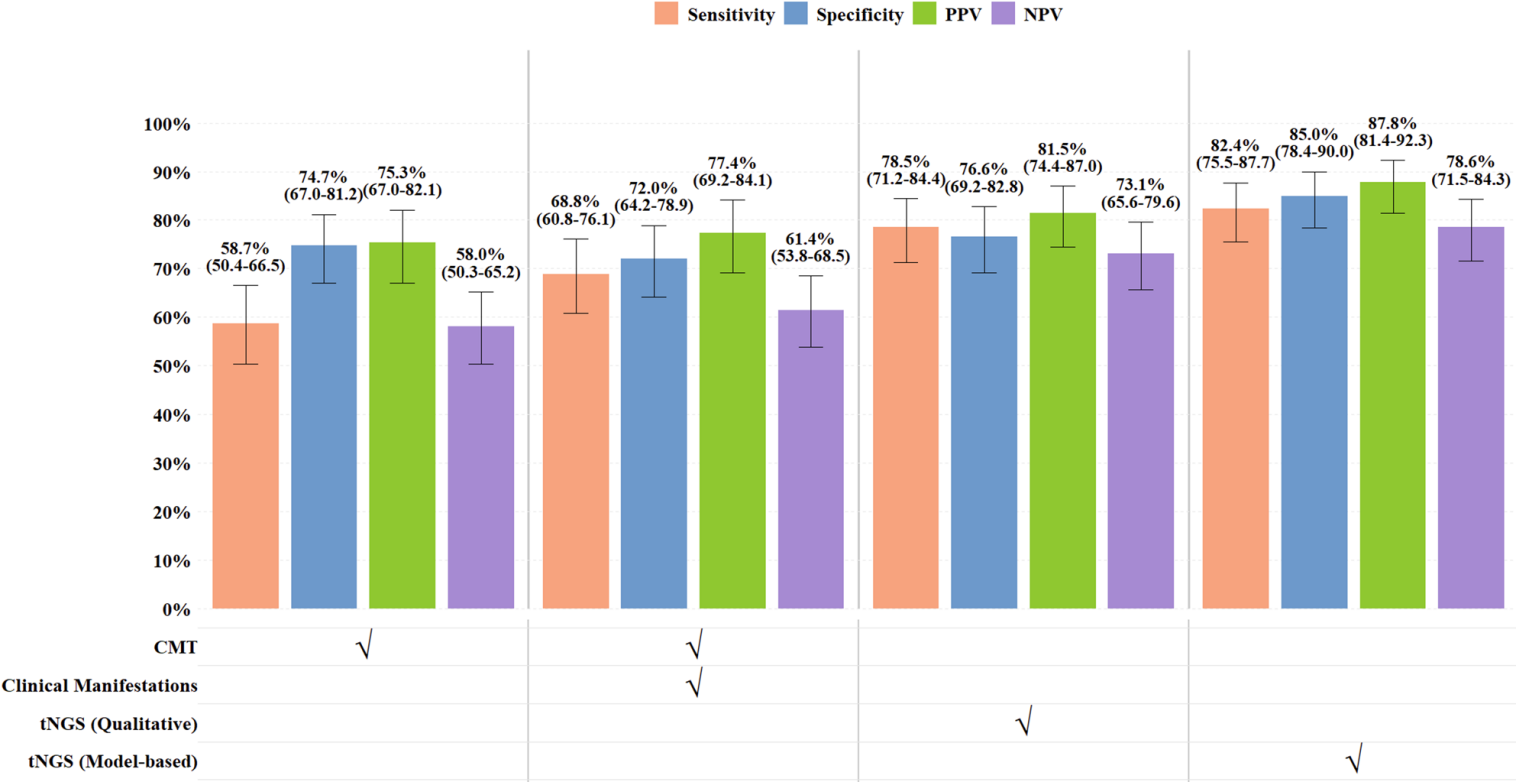
Diagnostic performance of CMT, CMT plus clinical manifestations, qualitative tNGS, and model-guided tNGS CMT, conventional microbiological testing; CMT plus clinical manifestations, CMT results integrated with clinical assessment (including symptoms, imaging features, and treatment response); Qualitative tNGS, identification based solely on microbial names without quantitative data; Model-guided tNGS, algorithm-based analysis of quantitative parameters

The diagnostic performances of CMT, CMT combined with clinical manifestations, tNGS (model-guided), and tNGS (qualitative assessment) were compared against reference pathogens identified by Expert Group A (Fig. 3). The sensitivity and specificity for conventional microbiological testing alone were 58.7% and 74.7%. Adding clinical manifestations to CMT resulted in a sensitivity of 68.8% and specificity of 72.0%. In comparison, qualitative tNGS achieved a sensitivity of 78.5% and a specificity of 76.6%, while the model-based algorithm demonstrated the highest diagnostic accuracy with a sensitivity of 82.4% and a specificity of 85.0%. Additionally, tNGS (model-guided) yielded the highest positive predictive value (PPV) (87.80%), and the highest negative predictive value (NPV) (78.64%).

### “False positives” and “false negatives” results of CMT and tNGS

**Fig. 4:**
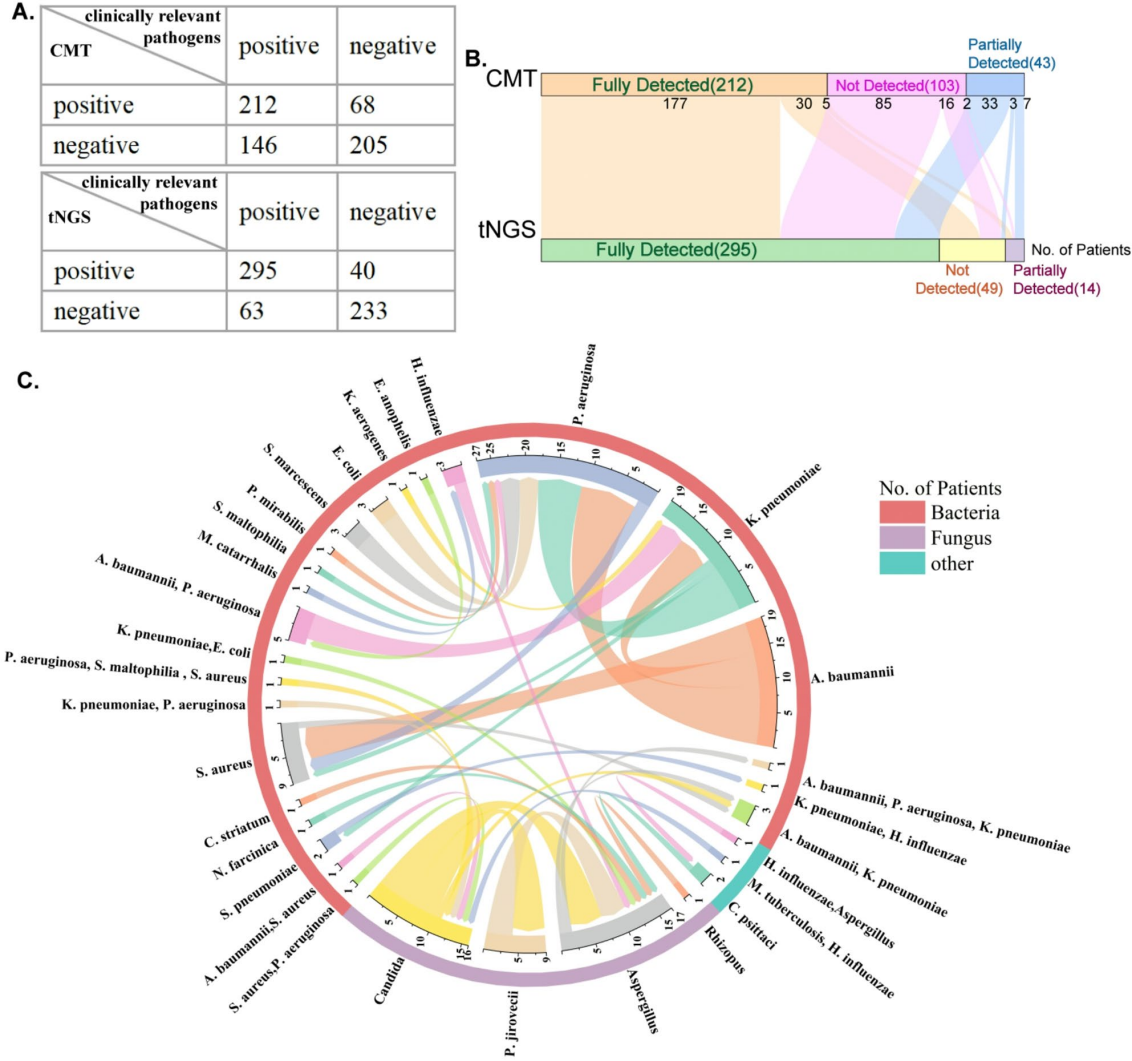
Comparative analysis of the Results detected by CMT and tNGS at patient level. **A** CMT and tNGS 2 × 2 Table of Patient-Level Detection. 2 × 2 contingency table showing the number of patients classified as positive or negative by CMT versus tNGS for clinically significant pathogens. **B** Sankey diagram illustrating the concordance of patient cases between tNGS and CMT methods. The flow bands represent the overlap between the two diagnostic approaches. **C** Chord diagram depicting co-infection cases. The circular segments show the number of patients infected with each pathogen (represented by different colors). The connecting chords indicate the number of patients harboring multiple pathogens

As illustrated in Fig. 4A, the patient-level 2 × 2 contingency tables demonstrated the comparative performance between CMT and tNGS against clinically significant pathogens identified by the expert panel. Prior to comparison, tNGS results were stratified using pre-validated models, thereby enabling evaluation of the models in clinical practice. False negative results refer to cases where the methods failed to detect clinically significant pathogens identified by the expert panel, while false positive results refer to cases in which the methods detected microbes not recognized as clinically significant pathogens. Using CMT, 68 cases were identified as false positive, and 146 cases were identified as false negative. In contrast, tNGS identified 40 false positive cases and 63 false negative cases.

**Fig. 5:**
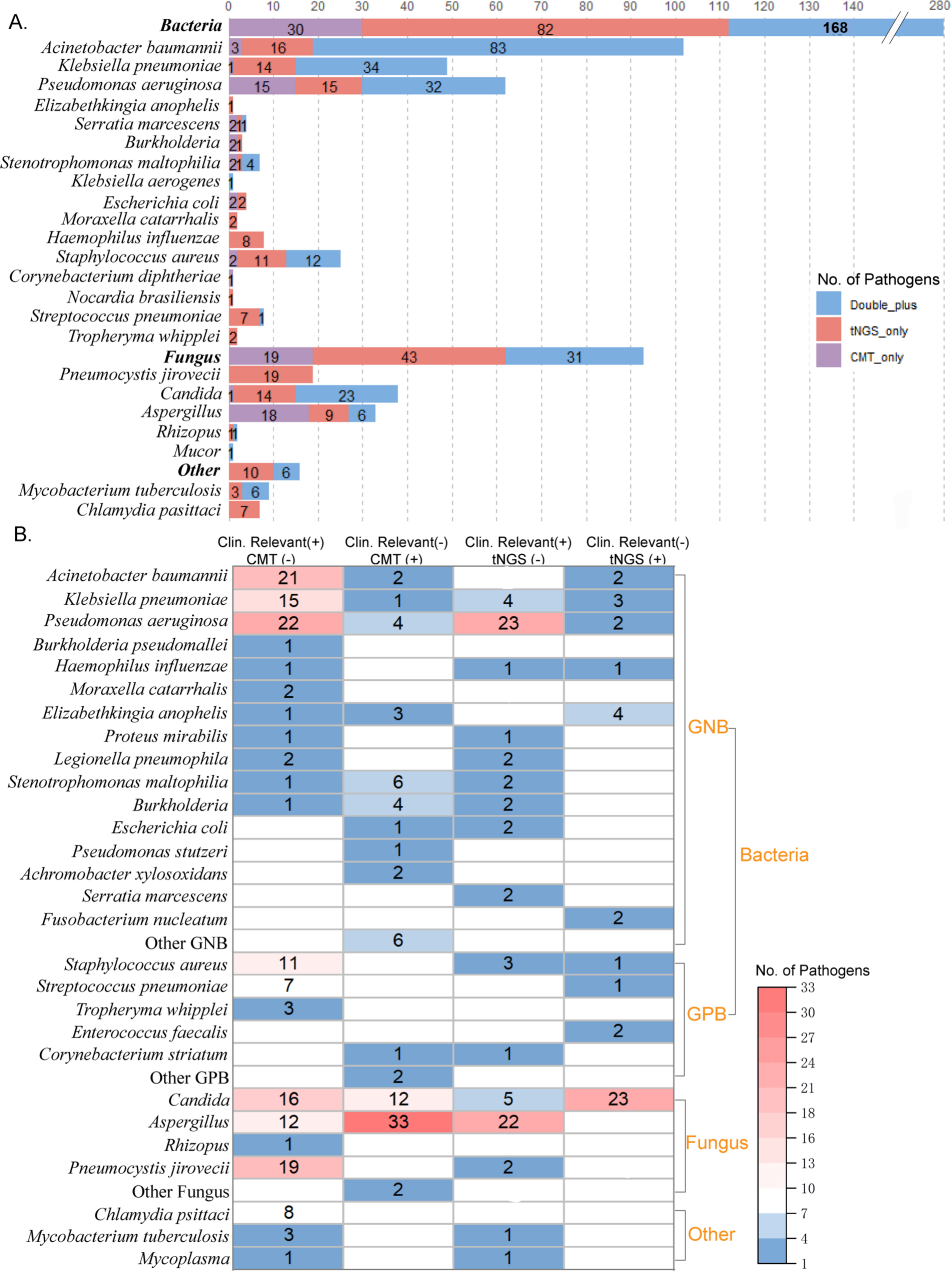
Comparative analysis of the Results detected by CMT and tNGS at pathogen level. **A** Stacked bar chart showing the distribution of detected pathogens by CMT and tNGS. The vertical axis lists different pathogens, while the horizontal axis indicates the number of detected pathogens. Red bars represent pathogens detected by tNGS only, purple bars represent pathogens detected by CMT only, and blue bars indicate pathogens detected by both methods. **B** Heatmap of Pathogen-Level False Positives and False Negatives. Heatmap illustrating, for each pathogen category (Gram-negative bacilli, Gram-positive bacilli, fungi and others), the frequency of false-positive and false-negative results by CMT and tNGS against the clinical relevant pathogens. Color intensity reflects misclassification counts (deep red = higher)

The pathogen-level heatmap analysis (Fig. 5B) provides a comprehensive visualization of the comparative performance between tNGS and CMT, displaying false positive and false negative patterns across different microbial taxa for individual microbial species. Hierarchical clustering analysis revealed distinct groupings of microbes based on shared detection profiles between the two methods.

The Clin. Significant(+) CMT(–) column highlights specific clinically significant pathogens that were missed by CMT, with majority of them being Gram-negative bacteria such as *Pseudomonas aeruginosa* (22 missed cases), *Acinetobacter baumannii* (21 missed cases), and *Klebsiella pneumoniae* (15 missed cases). Additionally, CMT failed to identify a substantial number of Gram-positive bacteria, including 11 cases of *Staphylococcus aureus*. Fungal pathogens were also frequently missed by CMT, with *Candida* species (16 missed cases), *Pneumocystis jirovecii* (19 missed cases), and *Aspergillus* species (12 missed cases) being the most frequently missed. The Clin.Significant(+) tNGS(–) column demonstrates clinically significant pathogens missed by tNGS, with *Pseudomonas aeruginosa* (23 missed cases) and *Aspergillus* species (22 missed cases) being the most prominent.

As shown in the Clin. Significant(–) CMT(+) column, CMT isolated microbes that were considered as clinically insignificant (false positives), particularly environmental fungi, including *Aspergillus* species (33 false positives) and *Candida* species (12 false positives). Similarly, the Clin. Significant(–) tNGS(+) column indicates that tNGS generated false positives with specific microbes, primarily environmental fungi and commensal bacteria, including *Elizabethkingia anophelis* (4 false positives) and *Candida* species (23 false positives).

Notably, CMT and tNGS exhibited significant overlap in their limitations for detecting certain pathogens. Both methods failed to detect (false negative) significant number of *Pseudomonas aeruginosa* (22 cases for CMT vs. 23 cases for tNGS, respectively) and *Aspergillus* species (12 cases for CMT vs. 22 cases for tNGS, respectively). In contrast, both methods identified clinically insignificant microbes (false positives), with *Candida* species being the most frequent detected false positives (12 cases for CMT vs. 23 cases for tNGS). These results highlight shared diagnostic challenges associated with *Pseudomonas aeruginosa*, *Aspergillus* species, and *Candida* species across both methods.

#### Detection of clinically significant pathogens between CMT and tNGS in LRTI

We evaluated the detection capability of CMT compared with tNGS in LRTI. tNGS results were first stratified using pre-validated models before comparative analysis. The Sankey diagram demonstrates the overall concordance at the patient level between these two methods, showing both overlapping and unique patient detection results (Fig. 4B). Among these patient cases, tNGS fully detected all clinically significant pathogens identified by expert panel A in 295 patients, while CMT fully detected all clinically significant pathogens in 212 patients. Notably, both methods successfully identified all clinically significant pathogens in 177 patients. Additionally, tNGS partially detected clinically significant pathogens in 14 patients, meaning that not all clinically significant pathogens identified by the expert panel A were detected, while CMT partially detected clinically significant pathogens in 43 patients. Furthermore, tNGS failed to detect pathogens in 49 patients, while CMT failed in 103 patients, with 16 patients showing no pathogen detection by either method. The clinical utility of rapid tNGS pathogen identification is exemplified through two cases of psittacosis and tuberculosis (Figure S6A, S6B), where tNGS results were correlated with culture findings throughout hospitalization.

Further analysis of specific pathogens revealed differences in detection capabilities between the two methods (Fig. 5A). tNGS demonstrated superior detection for *Acinetobacter baumannii*, *Klebsiella pneumoniae*, *Haemophilus influenzae*, *Staphylococcus aureus*, *Streptococcus pneumoniae*, *Pneumocystis jirovecii*, *Candida* species, and *Chlamydia psittaci*, while CMT showed better detection for *Aspergillus* species. Considering that some pathogens are difficult to cultivate on culture media, 63 tNGS positive cases caused by pathogens that are not usually grown on culture have been summarized in Table S5. This includes fastidious bacteria, Mycobacterium tuberculosis, atypical pathogens and non-cultivable fungi. tNGS exhibited a substantially higher detection rate for fastidious bacteria, atypical pathogens, and non-cultivable fungi in comparison with CMT. tNGS detected 100% of *Haemophilus influenzae*, *Legionella pneumophila*, *Mycoplasma pneumoniae*, *Chlamydia psittaci*, and *Pneumocystis jirovecii*, as well as 90% of *Mycobacterium tuberculosis* and 87.5% of *Streptococcus pneumoniae*. Only 60% of *Legionella pneumophila* cases were detected by urine antigen, and only 60% of *Mycobacterium tuberculosis* cases were detected by PCR. The findings emphasise the clear advantage of tNGS in identifying pathogens that are not usually grown on culture.

To further elucidate the differential performance of CMT and tNGS in the detection of *Aspergillus*, we conducted a species-level analysis of the 33 true positive cases (see Table S6). Among the 24 true positive *A. fumigatus* cases, tNGS identified only 8 (33.3%). This detection rate was notably lower than that of CMT (75.0%) and also lower than GM alone (45.8%). Due to the limited number of cases for *A. flavus* (*n* = 5), *A. terreus* (*n* = 3), and *A. niger* (*n* = 1), the observed differences in detection rates between GM and tNGS are not statistically meaningful. Additionally, our cohort was predominantly non-neutropenic. All GM-positive results were obtained from BALF, whereas serum GM was negative in every patient.

Among 358 patients with LRTI, 77 were identified as having co-infections. The pathogens responsible for these co-infections were analyzed and illustrated in Fig. 4C. A variety of pathogen combinations were observed, with *Acinetobacter baumannii* and *Pseudomonas aeruginosa* (*n* = 9) being the most common combination, followed by *Acinetobacter baumannii* and *Klebsiella pneumoniae* (*n* = 5). This finding suggests that certain pathogens tend to occur simultaneously in LRTI patients.

### Performance of tNGS in antimicrobial resistance prediction

**Fig. 6:**
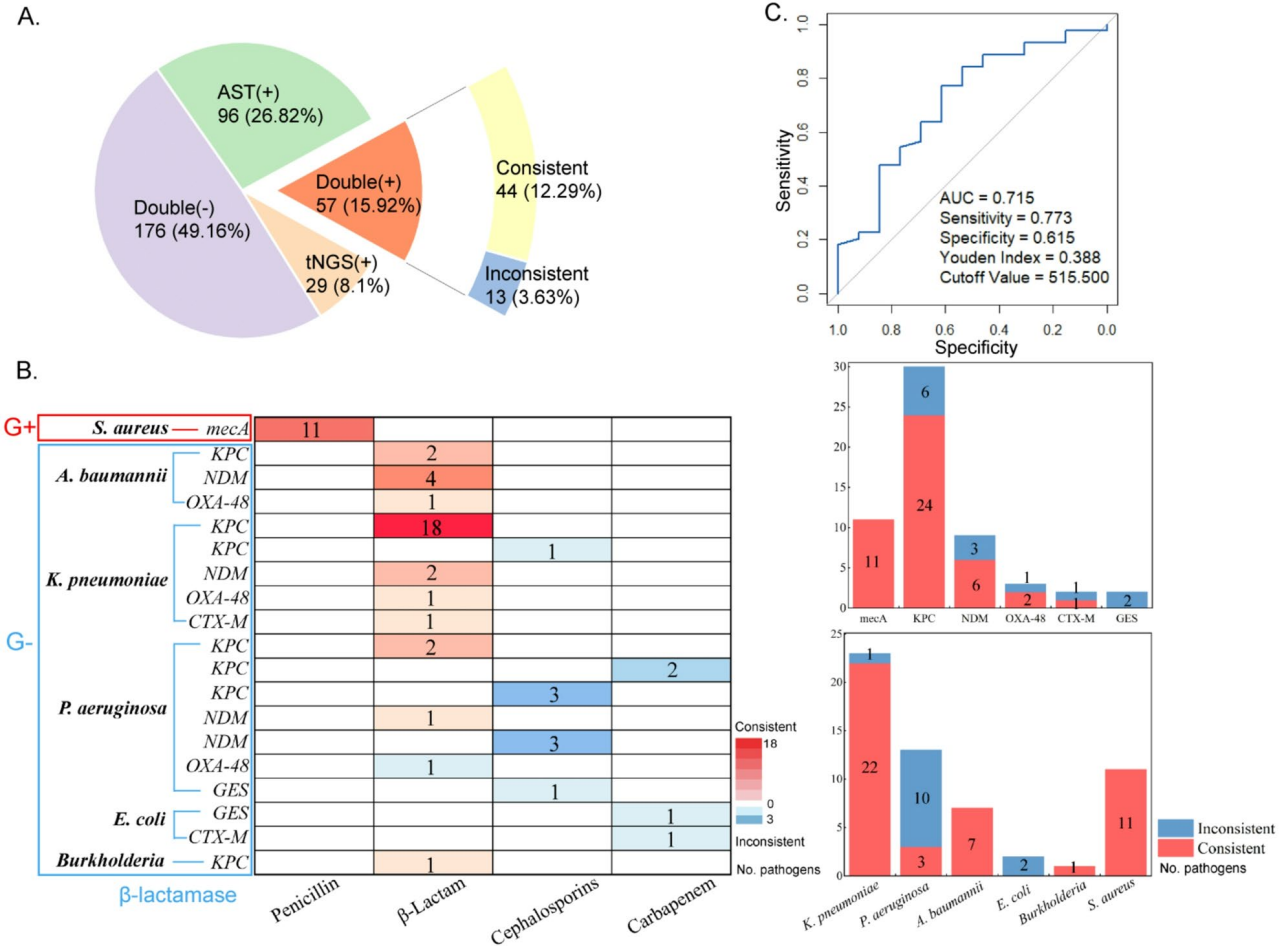
Antimicrobial resistance gene detection by tNGS compared to AST as the reference standard. **A **Pie chart illustrating distribution of drug-resistant genes detected at patient level in comparison with AST results**. B **Heat map demonstrating the correlation between drug resistance genes and phenotypes in bacteria, with red blocks indicating concordance and blue blocks indicating discordance between AST and tNGS results. Case counts are provided within the blocks. The adjacent stacked bar charts further detail this correlation, categorized by gene and bacterial species. **C **ROC analysis of drug-resistant genes detected by tNGS for phenotype prediction using RPKM values. AUC = 0.715; sensitivity = 0.773; specificity = 0.615; Youden index = 0.388; optimal cutoff value = 515.500

To demonstrate the potential of tNGS in predicting antimicrobial resistance, we further analyzed the concordance between resistance genes identified by tNGS and AST results in 358 patients with LRTI (Fig. 6). The overall distribution analysis (Fig. 6A) revealed that AST identified phenotypic resistance in 153 patients. Interestingly, tNGS identified resistance genes in 29 phenotypically susceptible (no resistance with AST) isolates. Meanwhile, 176 patients showed no evidence of resistance by either method. For the 57 patients where resistance was detected by both methods, concordance was observed in 44 cases, while the remaining 13 cases exhibited discordance between tNGS-detected resistance genes and phenotypic resistance patterns.

To further investigate the specific relationships between detected resistance genes and bacterial AST results, a heat map was generated to visualize their correlation (Fig. 6B), which illustrated the concordant and discordant results. Notably, several bacterial species, including *Klebsiella pneumoniae*, *Acinetobacter baumannii*, *Staphylococcus aureus*, and *Burkholderia species*, demonstrated higher consistency in resistance detection. However, considerable discrepancies were observed in *Escherichia coli* and *Pseudomonas aeruginosa*. Among the resistance genes, the detection of *KPC*, *NDM*, *OXA-48*, and *mecA* correlated well with AST results, whereas the detection of *GES* exhibited poor consistency.

The predictive capability of RPKM for resistance phenotypes was evaluated through ROC curve analysis (Fig. 6C). The analysis yielded an area under the curve (AUC) of 0.715, with sensitivity and specificity of 0.773 and 0.615, respectively. The optimal cutoff value was established at 515.5 (Youden index = 0.388). These metrics indicate that RPKM values derived from tNGS demonstrate moderate diagnostic accuracy in predicting antimicrobial resistance phenotypes. Our findings highlight the potential utility of tNGS in antimicrobial resistance detection, while emphasizing the necessity for careful interpretation in clinical settings. The clinical significance of elevated *mecA* reads was exemplified by a fatal MRSA infection case presenting with 10,285 *mecA* reads (Figure S6C).

## Discussion

LRTIs represent a significant global health burden [18], with particularly high mortality rates of 30.8% [19]. Early identification of causative pathogens and implementing targeted antimicrobial treatments are crucial for optimal clinical outcomes [20]. Although conventional cultures are still considered as the gold standard for identifying causative pathogens and guiding antimicrobial treatment through susceptibility tests, they are inherently time-consuming and inefficient [21]. NGS has emerged as a rapid diagnostic tool enabling precise pathogen identification and optimized antimicrobial therapy in patients with LRTI [22]. Despite its advantages, mNGS faces significant challenges, including high cost, technical complexity, and the ambiguous clinical significance for some detected microorganisms [23, 24]. tNGS presents a cost-effective alternative at approximately $170 per test—one-quarter of mNGS costs—while maintaining comparable detection efficiency through targeted genomic analysis [25]. However, tNGS interpretation in LRTIs remains heavily reliant on multidisciplinary collaborative assessment with experienced practitioners, with a lack of standardization that significantly limit its clinical implementation.

Recent studies have primarily compared tNGS to culture-based methods for pathogen detection in LRTIs, demonstrating its superior diagnostic performance [26]. Gaston et al. compared the pathogen detection capabilities of mNGS and tNGS in bronchoalveolar lavage fluid (BALF), revealing that tNGS achieved comparable sensitivity to mNGS while significantly reducing sequencing costs [27]. Other investigations have highlighted the utility of tNGS in diagnosing polymicrobial infections and infections among immunocompromised populations [28]. However, despite these advancements, current tNGS studies exhibit marked inconsistency in result interpretation, with each research center employing distinct interpretive criteria that lack unified standards. This interpretive heterogeneity has resulted in significant variability in sensitivity and specificity across different studies. The absence of standardized interpretation protocols represents a fundamental barrier to establishing tNGS as a reliable diagnostic modality in clinical settings. The implementation of quantitative interpretation models utilizing objective tNGS parameters could substantially reduce this interpretive variability, establish reproducible diagnostic criteria, and facilitate the transition from research-based applications to standardized clinical diagnostics. Such evidence-based frameworks are essential for realizing the full clinical potential of this innovative technology.

Our multicenter, prospective study endeavors to advance tNGS clinical interpretation through two key innovations. First, we conducted the largest systematic evaluation of tNGS performance in LRTI diagnosis to date, integrating comprehensive conventional microbiological testing (CMT)—including smear microscopy, bacterial/fungal cultures, *Aspergillus* galactomannan assays, and *Mycobacterium tuberculosis* specific PCR—to establish a robust reference standard. Second, and most critically, we developed and validated the first objective, quantitative interpretation models for tNGS in LRTIs. By leveraging tNGS-derived parameters (RPKM and pathogen copy number) in a training cohort of 420 patients and independently validating these models in 211 patients, we established pathogen-specific thresholds that reliably distinguish clinically significant pathogens from background commensals and contaminations. This methodological advance transforms tNGS from a qualitative screening tool into a quantitative diagnostic platform, enabling standardized interpretation without requiring specialized expertise.

Our study establishes tNGS-derived RPKM (sequence density normalization) and pathogen copy number (direct burden quantification) as robust discriminators of clinically significant pathogens in LRTI. We developed nomograms that achieved high diagnostic accuracy across microbial groups, with the overall bacterial model (incorporating all detected bacteria) showing excellent discrimination (AUC > 0.92). Given their high prevalence in ICU respiratory infections and substantial representation in our cohort, we further constructed pathogen-specific models for *Acinetobacter baumannii*, *Pseudomonas aeruginosa*, and *Klebsiella pneumoniae*—all demonstrating outstanding performance (AUC 0.845–0.972). Decision curve analysis (DCA) confirmed broad clinical utility for these bacterial models across nearly all threshold probabilities (0–99%), indicating robust readiness for clinical deployment. For fungal pathogens, while *Candida* species are typically considered colonizers that rarely cause lower respiratory tract infections, more stringent criteria are required to establish *Candida* associated LRTIs. Therefore, this study did not include the development of diagnostic models for *Candida* induced LRTIs. Additionally, insufficient case numbers precluded individual analyses of *Aspergillus* species and *Pneumocystis jirovecii*. To operationalize these advances, we developed an interactive web-based nomogram (https://brownvivian.shinyapps.io/RData/*)* enabling real-time implementation of the validated bacterial models.

Our study demonstrates that model-guided tNGS achieves superior diagnostic accuracy for LRTIs compared to CMT, CMT combined with clinical manifestations, and blinded qualitative assessment. To completely eliminate the potential incorporation bias arising from Expert Group A having access to tNGS quantitative data, we established Expert Group C, which relied solely on CMT results and clinical manifestations. The diagnostic performance of CMT alone had a sensitivity of 58.7% and a specificity of 74.7%. Adding clinical manifestations to CMT resulted in a sensitivity of 68.8% and specificity of 72.0%, reflecting real-world clinical judgment without tNGS data. Blinded qualitative tNGS (which used only microbial names without quantitative data) achieved a sensitivity of 78.5% and specificity of 76.6%. The lower specificity of blinded qualitative tNGS (76.6%) compared to model-guided tNGS (85.0%) reflects inherent clinical hesitation when interpreting pathogens without quantitative context, paradoxically suggesting that low-sequence detections may warrant consideration as potential pathogens. Notably, model-guided tNGS, which incorporates RPKM and copy number thresholds, yielded the highest diagnostic accuracy with a sensitivity of 82.4% and specificity of 85.0%. The fact that model-guided tNGS outperformed CMT combined with clinical manifestations group (sensitivity 82.4% vs. 68.8%; specificity 85.0% vs. 72.0%) demonstrates that the quantitative model’s advantage is not an overestimation of incorporation bias. Instead, it represents an improvement in diagnostic precision, enabling clinicians to distinguish true pathogens from colonizers or contaminants more accurately than with conventional methods or unaided clinical judgment.

The comparable diagnostic performance between model-guided tNGS and blinded qualitative assessment underscores the operational advantages of standardized quantitative interpretation for routine implementation. Automated RPKM and copy-number thresholding accelerates reporting timelines compared to multidisciplinary expert reviews, while predefined probability criteria ensure reproducibility across institutions with variable expertise. This scalability addresses a critical barrier to tNGS adoption in resource-limited settings.

Another strength of our study lies in the comprehensive evaluation of both accurately identified and erroneously classified pathogens across tNGS and CMT methodologies. While prior investigations predominantly focused on causative pathogens across these methods, our analysis extends this paradigm through a tripartite dissection of: (1) co-infection pathogen profiles, (2) false positive detections (environmental contaminants or commensals misclassified as pathogens), and (3) false negative omissions (clinically significant pathogens undetected due to technical limitations). This approach, systematically applied to 631 LRTI patients, uncovers how methodological biases—such as the dependency on pathogen viability for CMT and the susceptibility to environmental signal interference for tNGS—fundamentally distort microbial landscape characterization. By delineating these biases, our findings provide a framework for recalibrating diagnostic algorithms to mitigate context-dependent errors.

The co-detection of multiple pathogens in 77 patients (12.2%) exposes complex microbial interactions within the lower respiratory tract. Biofilms formed by Pseudomonas aeruginosa and Acinetobacter baumannii in co-infection scenarios often exhibit enhanced drug resistance, a phenomenon facilitated by the shared environment in the ICU where biofilms can promote cross-species colonization [29]. Furthermore, the co-existence of Klebsiella pneumoniae and Acinetobacter baumannii in biofilms is facilitated by shared antibiotic resistance genes, and these organisms often display cooperative interactions that promote their survival in hostile ICU environments [30]. Such genetic and metabolic synergism not only amplifies resistance phenotypes but also complicates therapeutic targeting. These findings underscore the imperative for ecological surveillance alongside pathogen detection to disrupt cooperative microbial networks in critical care settings.

We further analyze the comparative ability between tNGS and CMT at pathogen level. The discordance in pathogen detection between tNGS and CMT uncovers standard reference-specific diagnostic competencies shaped by microbial biology and technological constraints. tNGS demonstrated superior sensitivity for fastidious and intracellular pathogens, such as *Pneumocystis jirovecii* (19 vs. 0 true positives) and *Haemophilus influenzae* (8 vs. 0). These pathogens are either unculturable due to metabolic dependencie [31] or are suppressed by prior antibiotics (73% of our cohort received prior antibiotics). Conversely, CMT unexpectedly outperformed tNGS in *Aspergillus* detection (24 vs. 15 true positives), a paradox explained by multiple tests utilized for CMT: 63% of the Aspergillus cases identified by CMT were diagnosed using *Aspergillus* galactomannan antigen test. Galactomannan antigen test identifies a structural component of the fungal cell wall. This gives it several diagnostic advantages compared to tNGS. *Aspergillus* galactomannan antigen test detects fungal cell wall components and does not require cell wall disruption. tNGS requires efficient fungal cell wall disruption, which is technically challenging. Aspergillus has a thick, melanized cell wall. The difficulty of lysing the *Aspergillus* cell wall leads to underperformance of DNA-based methods [32]. This highlights that fungal diagnosis requires multiple testing approach, as no single method universally overcomes biological barriers.

The systematic comparison of misclassified pathogens revealed critical methodological divergences between tNGS and CMT, rooted in their distinct technical and biological limitations. CMT demonstrated high false negative rates for fungal pathogens—particularly *Candida* spp. (16 undetected cases) and *Pneumocystis jirovecii* (19 cases)—a pattern attributable to the inherent limitations of culture-dependent methods. Underdetection of *Candida* species stems from the low fungal burden and uneven distribution of pathogens in clinical specimens, as well as suppression by empirical antifungal therapy [33], while *Pneumocystis* detection is hampered by the absence of routine immunofluorescence staining in most clinical laboratories and the absence of standard culture methods [34]. Similarly, fastidious bacteria like *Legionella pneumophila* and *Mycoplasma pneumoniae* were systematically missed by CMT due to the lack of growth on routine culture media for these pathogens [35, 36]. Conversely, tNGS exhibited its own vulnerabilities. It missed 23 cases of *Pseudomonas aeruginosa*, likely due to sequencing bias against GC-rich bacterial genomes during library preparation [37]. It also failed to detect 22 *Aspergillus* cases, possibly resulting from inefficient fungal cell wall lysis—a known technical challenge in fungal DNA extraction [38].

Our study reveals both concordant and divergent profiles between tNGS-based genotypic predictions and conventional AST phenotypic results. The overall agreement rate of 65.08% (49.16% double-negative, 15.92% double-positive) demonstrates tNGS’s potential to align with established AST for resistance screening. Importantly, tNGS identified an additional 8.1% resistance cases missed by AST, underscoring its complementary value in detecting cryptic resistance mechanisms. However, the 3.63% discordance highlights persistent challenges in directly translating genetic markers to clinical resistance phenotypes. Species-specific variations further illuminate this complexity: high concordance in *Klebsiella pneumoniae*,* Acinetobacter baumannii*, and *Staphylococcus aureus* aligns with these pathogens’ well-characterized resistance gene-phenotype relationships (e.g., *mecA*-mediated methicillin resistance). Only 3 (23.1%) showed consistent results between genotypic and phenotypic resistance testing for *Pseudomonas aeruginosa* among 13 total cases, while 10 (76.9%) exhibited discordance. *E. coli* demonstrated no consistent cases, with all 2 detected cases showing discordance between genotypic and phenotypic results. Discordance sources require mechanistic discussion. GES-1 of *Pseudomonas aeruginosa* does not possess significant carbapenem-hydrolyzing activity, whereas GES-5 does. Detection of a GES family gene without discrimination of its specific subtype may yield a positive genotype even when the isolate remains phenotypically susceptible to carbapenems [39]. Resistance gene silencing of *E. coli* has been reported [40]. Resistance genes can be transcriptionally inactive due to host chromosomal factors, leading to a susceptible phenotype despite a positive genotypic result. We plan to conduct dedicated experimental studies in future work.

Quantitative thresholds (RPKM) provided moderate predictive accuracy (AUC = 0.715; sensitivity = 0.773, specificity = 0.615), indicating that pathogen burden moderately correlates with resistance risk. These findings advocate for complementary implementation of tNGS and AST, combining tNGS’s rapid identification of genetic resistance markers with AST’s established capacity for phenotypic validation to optimize both timely intervention and antimicrobial stewardship precision.

Several limitations of our study warrant consideration. First, the lack of convincing and adequate diagnostic criteria impeded the establishment of *Candida* models, while insufficient case numbers prevented the development of prediction models for *Aspergillus* species and *Pneumocystis jirovecii*, highlighting the need for larger cohort studies in fungal pathogen detection. Second, while tNGS showed superior overall performance compared to CMT, both methods faced common challenges in detecting certain pathogens, particularly *Pseudomonas aeruginosa* and *Aspergillus* species, indicating potential technical barriers that require further investigation. Third, despite promising results in antimicrobial resistance prediction, the moderate predictive capability (AUC = 0.715) and variable concordance across different species highlight the continuing challenge of translating genetic markers into reliable phenotypic predictions. Fourth, the model-guided tNGS platform used in this study is based on the multiplex PCR enrichment method developed by Kingmed Medical Technology Co., Ltd. Our models may not be directly generalizable to other tNGS platforms without independent validation.

## Supplementary Information

Additional file 1: Table S1. Characteristics of 631 patients with suspected LRTI. Table S2. List of pathogens detected using tNGS. Table S3. List of drug-resistant genes detected using tNGS. Table S4. Multivariable Logistic Regression Analysis with Cluster-Robust Standard Errors. Table S5. Detection of fastidious bacteria, mycobacteria, atypical pathogens and non-cultivable fungi by tNGS versus CMT. Table S6. Species-level detection of Aspergillus by CMT and tNGS. Table S7. Care methods used for pathogens that are usually detected via culture independent methods. Table S8. Detailed concordance between sequencing-detected resistance genes and phenotypic results per drug class.

Additional file 2: Figure S1. A predictive nomogram for identifying clinically significant pathogens in patients with LRTI. Figure S2. Bacteria diagnostic model evaluation and validation. Figure S3. Acinetobacter baumannii diagnostic model evaluation and validation. Figure S4. Pseudomonas aeruginosa diagnostic model evaluation and validation. Figure S5. Klebsiella pneumoniae diagnostic model evaluation and validation. Figure S6. Integration of tNGS into infection management strategies for ICU patients with LRTI.

## Data Availability

The data and materials in the current study are available from the corresponding author upon reasonable request.
