# Supplementary material for: Quantitative interpretation models for targeted next-generation sequencing in lower respiratory tract infections: a multicenter prospective study
Source: Respir Res. 2026 May 9;27:269. doi: 10.1186/s12931-026-03690-7 (PMC13330432; doi:10.1186/s12931-026-03690-7)
Supplement: Supplementary file 1 — Additional file 1: Table S1. Characteristics of 631 patients with suspected LRTI. Table S2. List of pathogens detected using tNGS. Table S3. List of drug-resistant genes detected using tNGS. Table S4. Multivariable Logistic Regression Analysis with Cluster-Robust Standard Errors. Table S5. Detection of fastidious bacteria, mycobacteria, atypical pathogens and non-cultivable fungi by tNGS versus CMT. Table S6. Species-level detection of Aspergillus by CMT and tNGS. Table S7. Care methods used for pathogens that are usually detected via culture independent methods. Table S8. Detailed concordance between sequencing-detected resistance genes and phenotypic results per drug class. [file 12931_2026_3690_MOESM1_ESM.doc]

**Table S1.** Characteristics of 631 patients with suspected LRTI

|  | ALL (n=631) | LRTIs (n=358) | Non-LRTIs (n=273) | P value |
| --- | --- | --- | --- | --- |
| Age, median (IQR), years | 73 (59-77) | 70 (61.75-77) | 69 (57-76) | 0.015^a^ |
| Sex, female, n (%) | 210 (33.28) | 118 (32.96) | 92 (33.7) | 0.845^b^ |
| Any comorbidity, n (%) |  |  |  |  |
| Diabetes | 160 (25.36) | 90 (25.14) | 70 (25.64) | 0.886^b^ |
| Hypertension | 310 (49.13) | 181 (50.56) | 129 (47.25) | 0.411^b^ |
| Cardiovascular disease | 248 (39.3) | 146 (40.78) | 102 (37.36) | 0.384^b^ |
| Chronic lung disease | 141 (22.35) | 81 (22.63) | 60 (21.98) | 0.847^b^ |
| Tumor | 140 (22.19) | 78 (21.79) | 62 (22.71) | 0.782^b^ |
| Chronic liver disease | 70 (11.09) | 41 (11.45) | 29 (10.62) | 0.742^b^ |
| Hematological disease | 67 (10.62) | 33 (9.22) | 34 (12.45) | 0.191^b^ |
| Connective tissue disease | 54 (8.56) | 32 (8.94) | 22 (8.06) | 0.695^b^ |
| Renal disease | 96 (15.21) | 55 (15.36) | 41 (15.02) | 0.905^b^ |
| Inflammation biomarker, median (IQR) |  |  |  |  |
| WBC (^109/L) | 9.16 (6.41-12.19) | 9.51 (6.72-13.17) | 8.48 (6.06-11.41) | 0.004^a^ |
| NE (^109/L) | 7.61 (4.91-10.51) | 8.2 (5.4-111.26) | 6.84 (4.44-9.51) | <0.001^a^ |
| CRP (mg/L) | 73.04 (29.99-140.38) | 89.39 (36.55-157.98) | 54.05 (23.13-114.48) | <0.001^a^ |
| PCT (ug/L) | 0.37 (0.11-1.61) | 0.49 (0.14-2.47) | 0.29 (0.1-1.06) | <0.001^a^ |
| ESR (mm/h) | 43 (20-67) | 47 (23.25-69) | 36 (15-65) | 0.011^a^ |
| IL-6 (pg/mL) | 38.85 (15.95-90.58) | 43.48 (19.23-115.46) | 32.56 (11.84-71.41) | 0.001^a^ |
| Abnormality on chest radiograph, n (%) |  |  |  |  |
| Unilateral lesion | 35 (5.55) | 18 (5.03) | 17 (6.23) | 0.514^b^ |
| Bilateral lesion | 396 (62.76) | 225 (62.85) | 171 (62.64) | 0.957^b^ |
| Oxygenation index, median (IQR), mmHg | 251.72 (175-348.78) | 242.22 (174.58-345) | 260 (177-356.35) | 0.434^a^ |
| Hospital, median (IQR), days | 11 (7-17) | 12 (8-18) | 10 (7-15.5) | 0.006^a^ |
| ICU, median (IQR), days | 10 (7-15) | 10 (7-17) | 9 (6-14) | <0.001^a^ |
| Hospital death, n (%) | 135 (21.39) | 89 (24.86) | 46 (16.85) | 0.015^b^ |
| P Values were calculated by Manne Whitney U test(a) or Chi-Square test(b).  LRTI, lower respiratory tract infection; tNGS, targeted next-generation sequencing. | | | | |

**Table S2.** List of pathogens detected using tNGS

| Pathogen types | Pathogens | | |
| --- | --- | --- | --- |
| DNA viruses | Herpes simplex virus 1 | Herpes simplex virus 2 | Varicella Zoster Virus |
|  | Epstein-Barr virus | Cytomegalo Virus | Human herpes virus-6 |
|  | Human herpes virus-6A | Human herpes virus-6B | Human herpes virus-7 |
|  | Human mastadenovirus | Human mastadenovirus B | Human mastadenovirus C |
|  | Human mastadenovirus D | Human adenovirus 1 | Human adenovirus 2 |
|  | Human adenovirus B3 | Human adenovirus E4 | Human adenovirus 5 |
|  | Human adenovirus 6 | Human adenovirus 7 | Human adenovirus 11 |
|  | Human adenovirus 14 | Human adenovirus 21 | Human adenovirus 34 |
|  | Human adenovirus 35 | Human adenovirus 55 | Human adenovirus 57 |
|  | Human bocavirus 1 | Human bocavirus 2 | Human bocavirus 3 |
|  | Human bocavirus 4 | Human parvovirus B19 | BK polyomavirus |
|  | JC polyomavirus | WU Polyomavirus |  |
| RNA viruses | Influenza A virus | Influenza A(H1N1) virus | Influenza A(H1N1)pdm09 virus |
|  | Influenza A(H3N2) virus | Influenza A(H5N1) virus | Influenza A(H7N9) virus |
|  | Influenza B virus | Influenza B virus (B/Victoria) | Influenza B virus (B/Yamagata) |
|  | Influenza C virus | Rhinovirus | Rhinovirus A |
|  | Rhinovirus B | Rhinovirus C | Human respiratory syncytial virus A |
|  | Human respiratory syncytial virus B | Mumps orthorubulavirus | Measles morbillivirus |
|  | Rubella virus | Human respirovirus 1 | Human orthorubulavirus 2 |
|  | Human respirovirus 3 | Human orthorubulavirus 4 | SARS-CoV-2 |
|  | Human coronavirus 229E | Human coronavirus HKU1 | Human coronavirus NL63 |
|  | Human coronavirus OC43 | Human metapneumovirus | Enterovirus |
|  | Enterovirus A | Enterovirus B | Enterovirus C |
|  | Enterovirus D | Enterovirus A71 | Coxsackievirus A2 |
|  | Coxsackievirus B3 | Coxsackievirus A5 | Coxsackievirus A6 |
|  | Coxsackievirus A10 | Coxsackievirus A16 | Echovirus E18 |
|  | Echovirus E30 | Enterovirus D68 |  |
| Gram positive bacteria | non-tuberculous mycobacteria | *Mycobacterium avium complex* | *Mycobacterium avium* |
|  | *Mycobacterium intracellulare* | *Mycobacteroides abscessus complex* | *Mycobacteroides chelonae* |
|  | *Mycobacteroides abscessus* | *Mycobacterium xenopi* | *Mycobacterium gordonae* |
|  | *Mycobacterium kansasii* | *Mycobacterium scrofulaceum* | *Mycobacterium malmoense* |
|  | *Mycobacterium shimoidei* | *Mycobacterium szulgai* | *Mycobacterium asiaticum* |
|  | *Mycobacterium celatum* | *Mycobacterium simiae* | *Mycolicibacterium smegmatis* |
|  | *Mycolicibacterium fortuitum* | *Mycobacterium tuberculosis complex* | *Nocardia* |
|  | *Nocardia concava* | *Nocardia brasiliensis* | *Nocardia farcinica* |
|  | *Nocardia africana* | *Nocardia abscessus* | *Nocardia cyriacigeorgica* |
|  | *Nocardia terpenica* | *Nocardia otitidiscaviarum* | *Nocardia nova* |
|  | *Nocardia asteroides* | *Parvimonas micra* | *Rhodococcus hoagii* |
|  | *Staphylococcus aureus* | *Streptococcus pneumoniae* | *Streptococcus pyogenes* |
|  | *Streptococcus agalactiae* | *Streptococcus anginosus group* | *Streptococcus intermedius* |
|  | *Trueperella pyogenes* | *Tropheryma whipplei* | *Corynebacterium diphtheriae* |
| Gram negative bacteria | *Acinetobacter baumannii* | *Haemophilus influenzae* | *Moraxella catarrhalis* |
|  | *Acinetobacter junii* | *Legionella* | *Bordetella pertussis* |
|  | *Acinetobacter ursingii* | *Legionella pneumophila* | *Bordetella parapertussis* |
|  | *Enterobacter cloacae complex* | *Legionella longbeachae* | *Bordetella holmesii* |
|  | *Escherichia coli* | *Legionella bozemanae* | *Elizabethkingia anophelis* |
|  | *Klebsiella pneumoniae* | *Legionella micdadei* | *Elizabethkingia meningoseptica* |
|  | *Klebsiella variicola* | *Burkholderia mallei* | *Brucella* |
|  | *Klebsiella aerogenes* | *Burkholderia pseudomallei* | *Neisseria meningitidis* |
|  | *Klebsiella oxytoca* | *Burkholderia cepacia complex* | *Pasteurella multocida* |
|  | *Proteus mirabilis* | *Burkholderia cepacia* | *Bacteroides fragilis* |
|  | *Pseudomonas aeruginosa* | *Burkholderia cenocepacia* | *Fusobacterium nucleatum* |
|  | *Serratia marcescens* | *Burkholderia contaminans* | *Fusobacterium necrophorum* |
|  | *Stenotrophomonas maltophilia* | *Burkholderia multivorans* |  |
| Fungi | *Aspergillus fumigatus* | *Rhizopus* | *Candida albicans* |
|  | *Aspergillus flavus complex* | *Rhizopus oryzae* | *Candida tropicalis* |
|  | *Aspergillus niger complex* | *Rhizopus delemar* | *Candida parapsilosis* |
|  | *Aspergillus terreus complex* | *Rhizopus microsporus* | *Candida orthopsilosis* |
|  | *Cryptococcus neoformans* | *Rhizomucor* | *Candida] glabrata* |
|  | *Cryptococcus gattii* | *Rhizomucor pusillus* | *Pichia kudriavzevii* |
|  | *Pneumocystis jirovecii* | *Mucor irregularis* | *Trichosporon asahii* |
|  | *Talaromyces marneffei* | *Mucor racemosus* | *Meyerozyma guilliermondii* |
|  | *Lichtheimia* | *Scedosporium* | *Histoplasma capsulatum* |
|  | *Lichtheimia ramosa* | *Scedosporium apiospermum* | *Fusarium* |
|  | *Lichtheimia corymbifera* | *Scedosporium boydii* |  |
| Mycoplasma, Chlamydia, etc | *Mycoplasma pneumoniae* | *Chlamydia pneumoniae* | *Coxiella burnetii* |
|  | *Ureaplasma parvum* | *Chlamydia trachomatis* |  |
|  | *Ureaplasma urealyticum* | *Chlamydia psittaci* |  |

**Table S3. List of drug-resistant genes detected using tNGS**

| Drug resistance gene family | blaKPC |
| --- | --- |
|  | blaOXA-48 |
|  | mecA |
|  | blaNDM |
|  | blaIMP |
|  | blaVIM |
|  | blaSME |
|  | blaIMI |
|  | blaGES |
|  | blaGIM |
|  | blaSPM |
|  | A2063G |
|  | A2064G |
|  | A2067G |
|  | C2617G |

**Table S4**. Multivariable Logistic Regression Analysis with Cluster-Robust Standard Errors

| Predictor | beta | S.E. | Wald Z | P | OR (95% CI) |
| --- | --- | --- | --- | --- | --- |
| RPKM | 0.0001 | 0.0000 | 3.61 | 0.0003 | 2.53 (1.53 - 4.18) |
| COPY | 0.9575 | 0.1685 | 5.68 | < 0.0001 | 17.68 (6.57 - 47.62) |

Table S5. Detection of fastidious bacteria, mycobacteria, atypical pathogens and non-cultivable fungi by tNGS versus CMT

| Pathogen Category | Pathogen | Total True Positives (n) | Detected by tNGS (n, %) | Detected by CMT (n, %) | Detected by culture (n, %) |
| --- | --- | --- | --- | --- | --- |
| Fastidious bacteria | Haemophilus influenzae | 8 | 8 (100%) | 0 (0%) | 0 (0%) |
|  | Streptococcus pneumoniae | 8 | 7 (87.5%) | 1 (12.5%) | 0 (0%) |
|  | Legionella pneumophila | 5 | 5 (100%) | 3 (60%) | 0 (0%) |
| Mycobacterium tuberculosis | Mycobacterium tuberculosis complex | 10 | 9 (90.0%) | 6 (60.0%) | 0 (0%) |
| Atypical pathogens | Mycoplasma pneumoniae | 6 | 6 (100%) | 0 (0%) | 0 (0%) |
|  | Chlamydia psittaci | 7 | 7 (100%) | 0 (0%) | 0 (0%) |
| Non-cultivable fungi | Pneumocystis jirovecii | 19 | 19 (100%) | 0 (0%) | 0 (0%) |
| Total |  | 63 | 61 (96.8%) | 10 (15.9%) | 0 (0%) |

Table S6. Species-level detection of Aspergillus by CMT and tNGS

| Aspergillus Species | Total True Positives (Expert A) | Detected by CMT,n (%) | Detected by GM,n (%) | Detected by tNGS,n (%) |
| --- | --- | --- | --- | --- |
| A. fumigatus | 24 | 18 (75.0%) | 11 (45.8%) | 8 (33.3%) |
| A. flavus | 5 | 3 (60.0%) | 1 (20.0%) | 1 (20.0%) |
| A. terreus | 3 | 2 (66.7%) | 2 (66.7%) | 1 (33.3%) |
| A. niger | 1 | 1 (100.0%) | 1 (100.0%) | 1 (100.0%) |
| Total | 33 | 24 (72.7%) | 15 (45.5%) | 11 (45.5%) |

**Table S7.** Care methods used for pathogens that are usually detected via culture independent methods

| Pathogen | Standard of care methods | Implementation details |
| --- | --- | --- |
| Pneumocystis jirovecii | Periodic Acid-Schiff (PAS) stain and Gomori's methenamine silver (GMS) stain | The PAS stain and GMS stain were performed on patients with suspected Pneumocystis jirovecii infections |
| Aspergillus | Direct smear microscopy, calcofluor white (CFW) staining, GM-tests | All patients underwent smear microscopy, CFW stain and GM tests. GM tests was performed on blood and BALF samples |
| Rhizopus / Mucor | Direct smear microscopy, CFW stain | Smear microscopy and CFW stain were performed on all patients |
| Mycobacterium tuberculosis | Acid-fast stain, PCR | Acid-fast stain and PCR were performed on patients with suspected Mycobacterium tuberculosis  infections |
| Legionella pneumophila | Urine antigen test | Urine antigen test was performed for all patients with Legionella pneumophila serogroup 1 infection. |
| Chlamydia psittaci | None |  |

**Table S8.** Detailed concordance between sequencing-detected resistance genes and phenotypic results per drug class.

| Drug Class | Pathogen | Resistance Genes Detected | Consistent Cases, n (%) | Inconsistent Cases, n (%) | Total Cases, n |
| --- | --- | --- | --- | --- | --- |
| Penicillins | *Staphylococcus aureus* | mecA | 11 (100.0%) | 0 (0.0%) | 11 |
| β-Lactams | *Acinetobacter baumannii* | KPC, NDM, OXA-48 | 7 (100.0%) | 0 (0.0%) | 7 |
|  | *Klebsiella pneumoniae* | KPC, NDM, OXA-48, CTX-M | 22 (100.0%) | 0 (0.0%) | 22 |
|  | *Pseudomonas aeruginosa* | KPC, NDM, OXA-48, VIM | 3 (75.0%) | 1 (25.0%) | 4 |
|  | *Burkholderia* | KPC | 1 (100.0%) | 0 (0.0%) | 1 |
| Cephalosporins | *Klebsiella pneumoniae* | KPC | 0 (0.0%) | 1 (100.0%) | 1 |
|  | *Pseudomonas aeruginosa* | KPC, NDM, GES | 0 (0.0%) | 7 (100.0%) | 7 |
| Carbapenems | *Pseudomonas aeruginosa* | KPC | 0 (0.0%) | 2 (100.0%) | 2 |
|  | *Escherichia coli* | GES, CTX-M | 0 (0.0%) | 2 (100.0%) | 2 |
