# Supplementary material for: Quantitative interpretation models for targeted next-generation sequencing in lower respiratory tract infections: a multicenter prospective study
Source: Respir Res. 2026 May 9;27:269. doi: 10.1186/s12931-026-03690-7 (PMC13330432; doi:10.1186/s12931-026-03690-7)
Supplement: Supplementary file 2 — Additional file 2: Figure S1. A predictive nomogram for identifying clinically significant pathogens in patients with LRTI. Figure S2. Bacteria diagnostic model evaluation and validation. Figure S3. Acinetobacter baumannii diagnostic model evaluation and validation. Figure S4. Pseudomonas aeruginosa diagnostic model evaluation and validation. Figure S5. Klebsiella pneumoniae diagnostic model evaluation and validation. Figure S6. Integration of tNGS into infection management strategies for ICU patients with LRTI. [file 12931_2026_3690_MOESM2_ESM.doc]

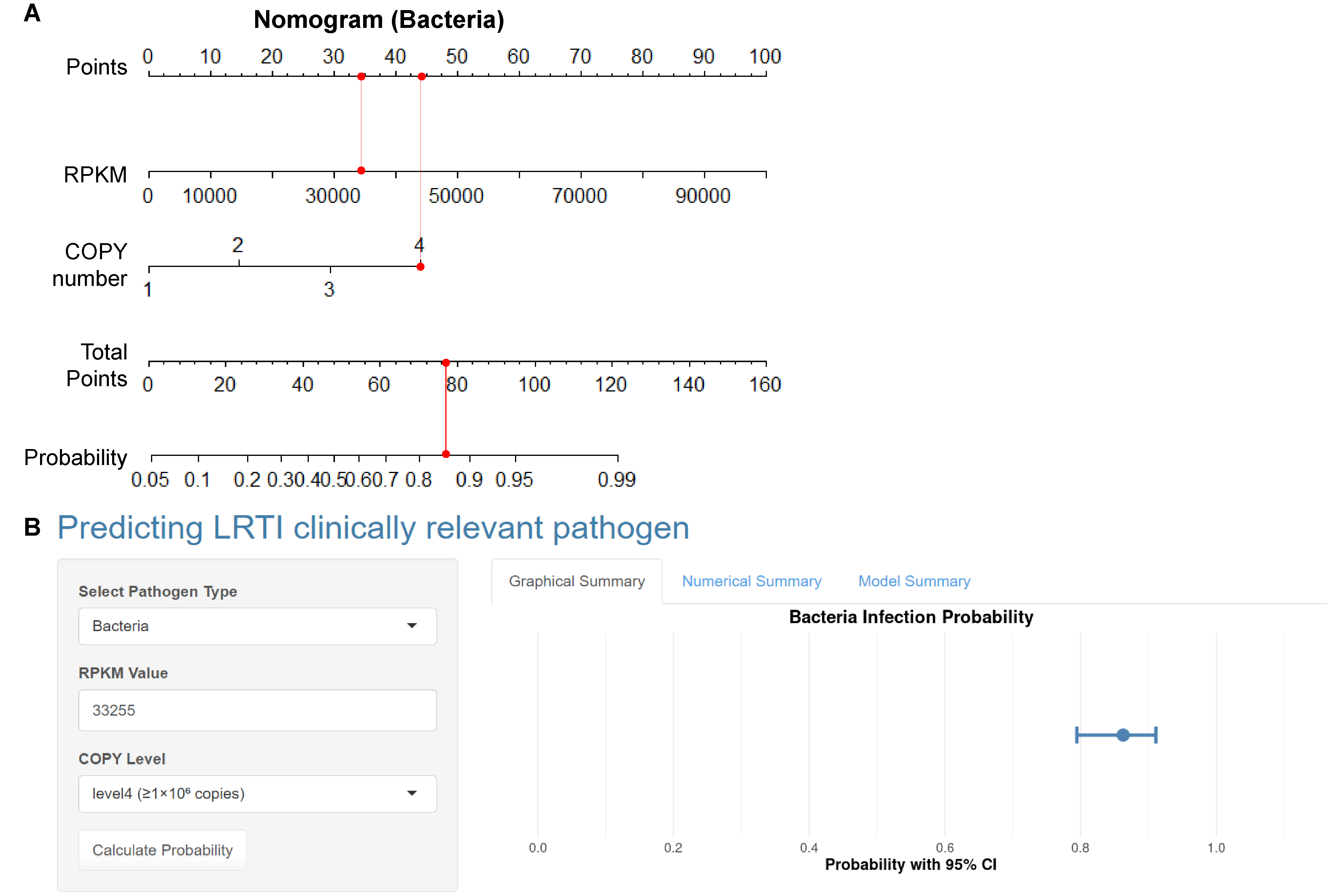


**Figure S1. A predictive nomogram for identifying clinically significant pathogens in patients with LRTI**

1. Construction and application of the nomogram. Individual points for each predictor variable can be determined by drawing a vertical line from the corresponding value to the "Points" axis. The sum of these individual points yields the total points, which correlates with the probability of clinically significant pathogen infection. To illustrate the nomogram's utility, we present a case example of a patient with bacterial infection: given an RPKM value of 33,255 and a COPY number at level 4 (≥1 × 10^6 copies), the calculated probability of bacterial infection was 86.26%.
2. Visual representation of the web-based interactive nomogram interface, accessible at https://brownvivian.shinyapps.io/RData/.


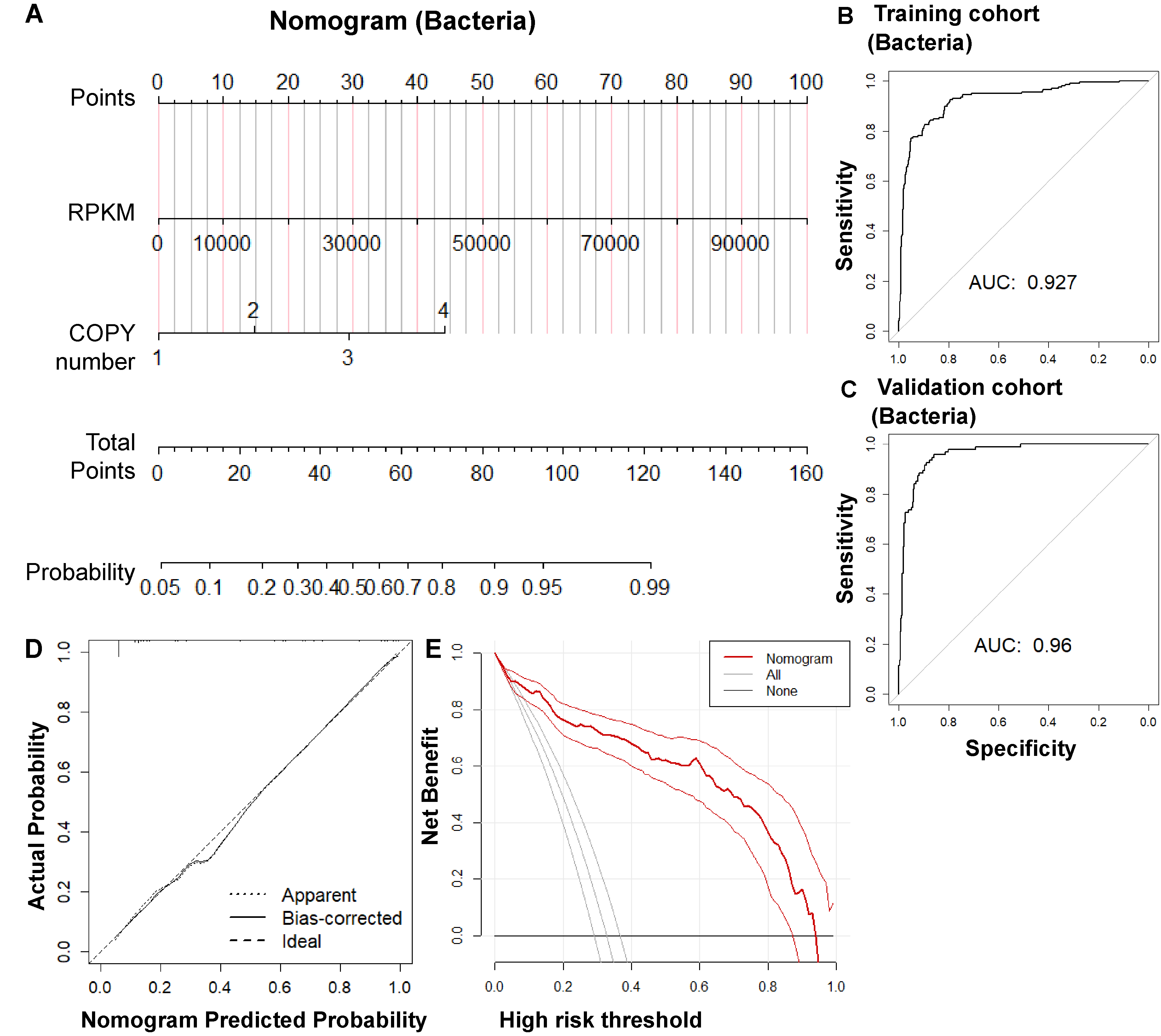


**Figure S2. Bacteria diagnostic model evaluation and validation**

1. Nomogram for predicting the efficacy of diagnostic model of bacteria in the training cohort.
2. ROC curve of the nomogram model in the training cohort. The AUC in training cohort was 0.927 (95% CI: 0.904–0.95).
3. ROC curve of the nomogram model in the validation cohort. The AUC in the validation cohort was 0.96 (95% CI: 0.942–0.979).
4. Calibration curve of the nomogram model in the training cohort; The x-axis shows the predicted probability of the LRTI, and the y-axis shows the observed probability of the LRTI. The ideal line means that the predicted and actual probabilities of the model agree perfectly. The apparent line indicates the actual performance of the prediction model in the training cohort. The bias-corrected line indicates the performance of the prediction model in the training cohort after the correction of the overfitting situation. The calibration curve and standard curve have a good fit in training cohort.
5. DCA curve of the nomogram model in the training cohort; The x-axis displays the probability threshold. The y-axis indicates the clinical benefit of the tNGS intervention. AUC, the area under curve; DCA, decision curve analysis. ROC, receive operator curve.


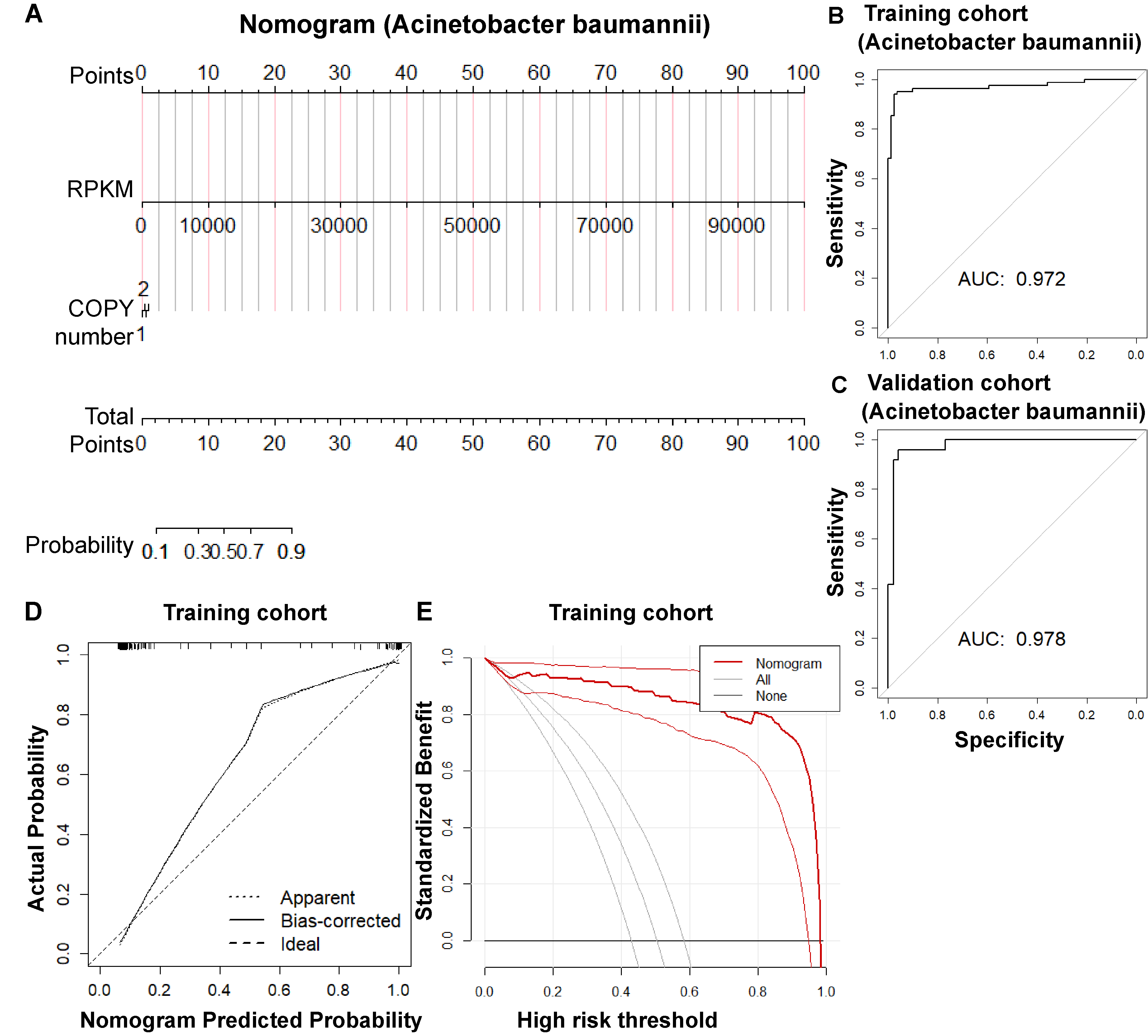


**Figure S3. Acinetobacter baumannii diagnostic model evaluation and validation**

1. Nomogram for predicting the efficacy of diagnostic model of Acinetobacter baumannii in the training cohort.
2. ROC curve of the nomogram model in the training cohort. The AUC in training cohort was 0.972 (95% CI: 0.945–0.999).
3. ROC curve of the nomogram model in the validation cohort. The AUC in the validation cohort was 0.978 (95% CI: 0.948–1).
4. Calibration curve of the nomogram model in the training cohort; The x-axis shows the predicted probability of the LRTI, and the y-axis shows the observed probability of the LRTI. The ideal line means that the predicted and actual probabilities of the model agree perfectly. The apparent line indicates the actual performance of the prediction model in the training cohort. The bias-corrected line indicates the performance of the prediction model in the training cohort after the correction of the overfitting situation. The calibration curve and standard curve have a good fit in training cohort.
5. DCA curve of the nomogram model in the training cohort; The x-axis displays the probability threshold. The y-axis indicates the clinical benefit of the tNGS intervention. AUC, the area under curve; DCA, decision curve analysis. ROC, receive operator curve.

**
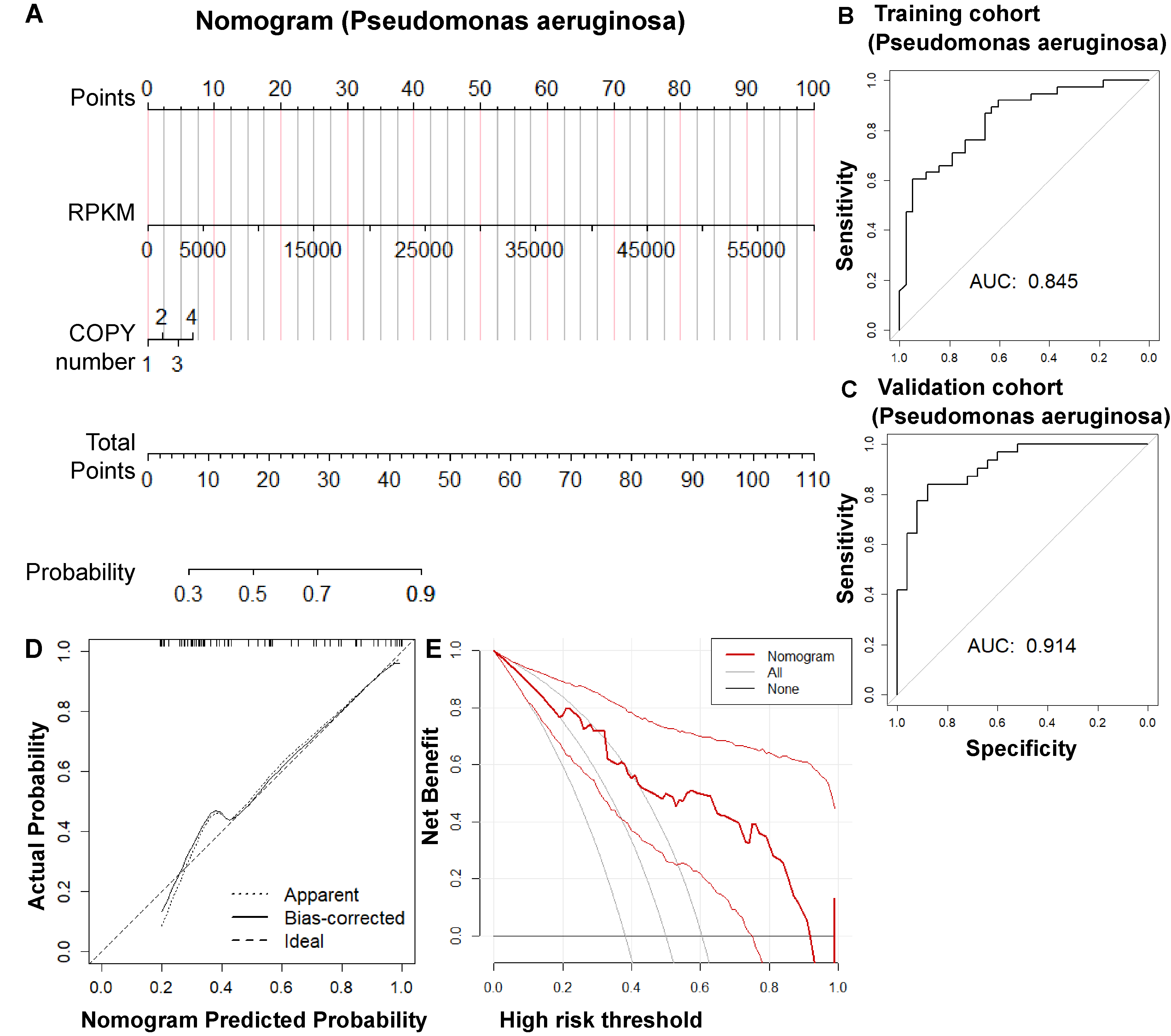
**

**Figure S4. Pseudomonas aeruginosa diagnostic model evaluation and validation**

1. Nomogram for predicting the efficacy of diagnostic model of Pseudomonas aeruginosa in the training cohort.
2. ROC curve of the nomogram model in the training cohort. The AUC in training cohort was 0.845 (95% CI: 0.758–0.932).
3. ROC curve of the nomogram model in the validation cohort. The AUC in the validation cohort was 0.914 (95% CI: 0.842–0.985).
4. Calibration curve of the nomogram model in the training cohort; The x-axis shows the predicted probability of the LRTI, and the y-axis shows the observed probability of the LRTI. The ideal line means that the predicted and actual probabilities of the model agree perfectly. The apparent line indicates the actual performance of the prediction model in the training cohort. The bias-corrected line indicates the performance of the prediction model in the training cohort after the correction of the overfitting situation. The calibration curve and standard curve have a good fit in training cohort.
5. DCA curve of the nomogram model in the training cohort; The x-axis displays the probability threshold. The y-axis indicates the clinical benefit of the tNGS intervention. AUC, the area under curve; DCA, decision curve analysis. ROC, receive operator curve.


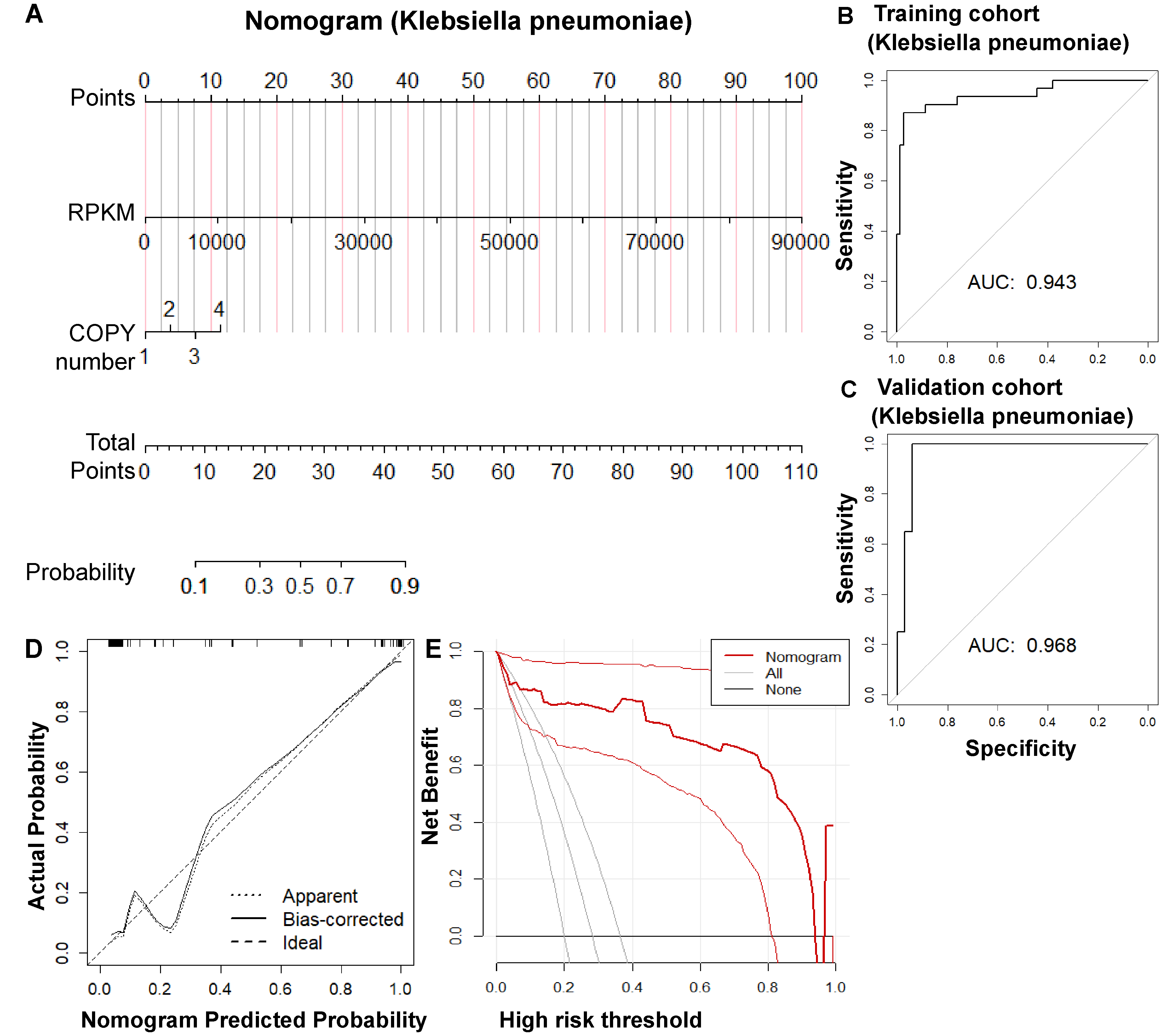


**Figure S5. Klebsiella pneumoniae diagnostic model evaluation and validation**

1. Nomogram for predicting the efficacy of diagnostic model of Klebsiella pneumoniae in the training cohort.
2. ROC curve of the nomogram model in the training cohort. The AUC in training cohort was 0.943 (95% CI: 0.887–0.998).
3. ROC curve of the nomogram model in the validation cohort. The AUC in the validation cohort was 0.968 (95% CI: 0.919–1).
4. Calibration curve of the nomogram model in the training cohort; The x-axis shows the predicted probability of the LRTI, and the y-axis shows the observed probability of the LRTI. The ideal line means that the predicted and actual probabilities of the model agree perfectly. The apparent line indicates the actual performance of the prediction model in the training cohort. The bias-corrected line indicates the performance of the prediction model in the training cohort after the correction of the overfitting situation. The calibration curve and standard curve have a good fit in training cohort.
5. DCA curve of the nomogram model in the training cohort; The x-axis displays the probability threshold. The y-axis indicates the clinical benefit of the tNGS intervention. AUC, the area under curve; DCA, decision curve analysis. ROC, receive operator curve.


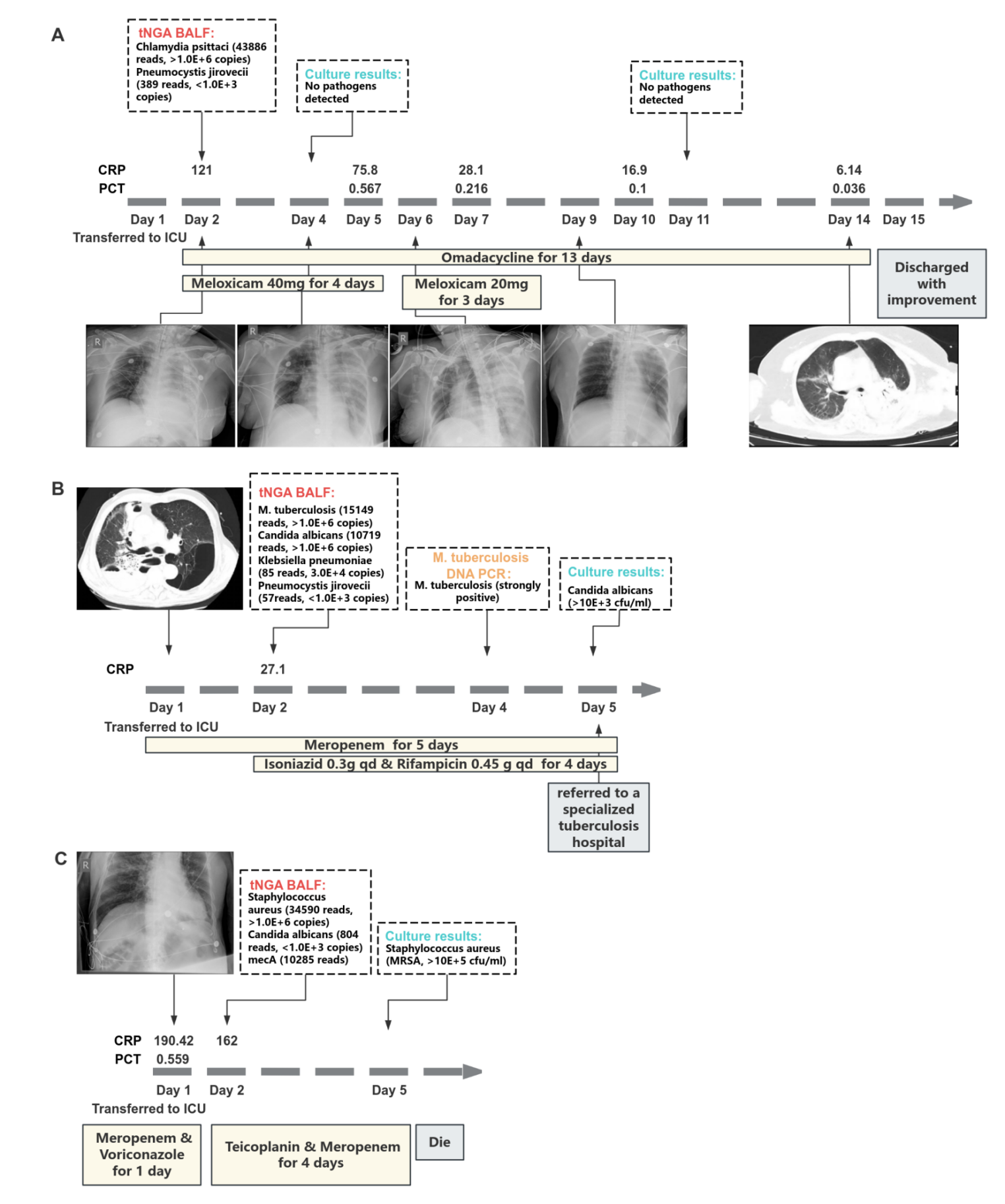


**Figure S6. Integration of tNGS into infection management strategies for ICU patients with LRTI**

1. A 59-year-old female patient diagnosed with psittacosis.
2. A 77-year-old male patient diagnosed with tuberculosis.
3. A 85-year-old male patient diagnosed with MRSA.

CRP: C-reactive protein; PCT: Procalcitonin; CFU: colony-forming unit.
